# Clinical characteristics, genetic spectrum and therapeutic effects of 51 male patients with idiopathic hypogonadotropic hypogonadism from southern China

**DOI:** 10.1186/s13023-025-04050-2

**Published:** 2025-11-12

**Authors:** Huiying Sheng, Cuili Liang, Jing Cheng, Huazhen Liu, Xiaojian Mao, Xiuzhen Li, Duan Li, Zhikun Lu, Yanna Cai, Xueying Su, Liyu Zhang, Wen Fu, Jinhua Hu, Wei Jia, Guochang Liu, Wen Zhang, Li Liu, Yunting Lin

**Affiliations:** 1 Department of Genetics and Endocrinology, Guangzhou Women and Children’s Medical Center, Guangzhou Medical University, Guangzhou, 510623 China; 2 Department of Pediatric Urology, Guangzhou Women and Children’s Medical Center, Guangzhou Medical University, Guangzhou, 510623 China

**Keywords:** Idiopathic hypogonadotropic hypogonadism, Clinical characteristics, Imaging findings, Hormonal features, Genetic spectrum, Therapeutic effects

## Abstract

**Background:**

Idiopathic hypogonadotropic hypogonadism (IHH) is a set of rare diseases characterized by abnormal sexual development with clinical heterogeneity and genotypic complexity. This study aims to investigate the phenotypic and genotypic characteristics of male IHH in southern China, and evaluate the therapeutic effects of current treatments.

**Methods:**

Fifty-one male IHH patients from southern China were enrolled in this study. Their clinical, imaging, hormonal and genetic findings were analyzed retrospectively.

**Results:**

In this study, the most common causative gene of IHH was *FGFR1* (45.10%), followed by *ANOS1* (21.57%) and *CHD7* (17.65%). Forty-five different variants, including 22 known and 23 novel variants, were found. The mean age at diagnosis was 7.84 ± 5.89 years, the most common clinical phenotype was micropenis (98.04%), the most frequent imaging feature was abnormal ultrasound of sexual glands (86.84%), and the most representative biochemical manifestations were low basal luteinizing hormone (LH) and testosterone (98.04% and 100.00%, respectively). Age-phenotype and genotype-phenotype correlations were observed in this cohort. The penile length, testicular volume, basal testosterone, and the proportion of patients with low basal inhibin B were associated with age. Most patients with *ANOS1* variant had a family history, impaired olfactory function, and much lower basal anti-mullerian hormone (AMH), whereas patients with *CHD7* variant were younger, presented CHARGE phenotypes, and had higher basal follicle-stimulating hormone (FSH) and LH. Moreover, 34 patients were treated with different strategies for 2.75 ± 1.82 years. After treatment, the penile length, and the levels of FSH, LH and testosterone increased significantly.

**Conclusions:**

Our study adds 51 southern Chinese male patients, and expands the mutational spectrum for IHH. Our cohort suggests that a combination of clinical, biochemical and genetic criteria will facilitate early diagnosis. Our work also highlights the differentially diagnostic values of family history, impaired olfactory function, CHARGE features, and basal AMH, FSH and LH in distinguishing different molecular bases of IHH.

**Supplementary Information:**

The online version contains supplementary material available at 10.1186/s13023-025-04050-2.

## Introduction

Idiopathic hypogonadotropic hypogonadism (IHH), also known as isolated hypogonadotropic hypogonadism or isolated gonadotropin-releasing hormone (GnRH) deficiency, is a set of rare diseases caused by defects in the hypothalamic-pituitary-gonadal axis, resulting in insufficient GnRH but normal levels of other pituitary hormones [1, 2]. The prevalence of IHH is estimated to be 1–10 per 100,000 births with males having a 2.6-fold to 5-fold higher prevalence than females [3–6].

The typical clinical manifestations of IHH include lack of sexual development, small genitalia, small sexual glands and delayed puberty, accompanying by biochemical features of inappropriately low levels of gonadotropins, luteinizing hormone (LH) and follicle-stimulating hormone (FSH), and sex steroids [3, 7]. According to the olfactory function, IHH can be divided into two groups: (1) Kallmann syndrome, associated with anosmia or hyposmia and accounting for 60% of patients; and (2) normosmic IHH, responsible for the remaining 40% of patients [1, 3].

Clinical variability of expression and penetrance has been observed in patients with IHH due to the genetic heterogeneity [1, 8]. The molecular mechanisms underlying IHH are diverse. To date, more than 30 different causative genes have been reported to cause IHH with inheritance patterns including X-linked recessive, autosomal dominant and autosomal recessive [9–11]. Among them, *ANOS1*, *CHD7*, *FGFR1*, *GNRHR*, *PROK2*, *PROKR2*, *IL17RD*, *SOX10*, *TAC3*, *TACR3*, *FGF8*, *KISS1* and *KISS1R* gene are the most frequent molecular bases [3, 9]. However, a genetic etiology has been identified in only about 50% of IHH cases, whereas the cause remains unknown in the others [2, 4, 8, 12].

The treatment of IHH includes steroid replacement therapy, human chorionic gonadotropin (HCG) therapy, combined gonadotropin therapy, pulsatile GnRH therapy, or sequential gonadotropin therapy [9, 13, 14]. Traditionally, patients with IHH are typically not diagnosed until late adolescence or early adulthood due to absence of pubertal development [12]. With advances in genetic testing, especially next-generation sequencing (NGS), the precise molecular diagnosis of IHH patients is facilitated, enabling timely treatment and improving drug response [7, 13].

In the present study, we retrospectively analyzed the clinical, imaging, hormonal and genetic findings of 51 male patients with IHH from southern China to study their clinical characteristics and genetic spectrum, and assess the therapeutic effects of current treatments.

## Materials and methods

### Patients

Fifty-one male IHH patients from 48 unrelated families were enrolled in Guangzhou Women and Children’s Medical Center from November 2015 to December 2024. All subjects were of Han ethnicity and from southern China. None of the parents were consanguineous.

The inclusion criteria were as follows:1) clinical signs of hypogonadism (including micropenis and/or cryptorchidism) for boys younger than 14 years of age; clinical signs of hypogonadism and no development of secondary sexual characteristics for boys older than 14 years of age; 2) low levels of gonadotropins (LH < 1.0 IU/L) and/or testosterone (< 3.5 nmol/L) without abnormalities of other pituitary and adrenal hormones; 3) normal chromosome karyotype; 4) identification of pathogenic or likely pathogenic variants in the causative genes of IHH [15]. Only patients who meet all four criteria were included in the IHH cohort.

### Clinical information

Medical history and clinical data were collected and evaluated by clinicians. Physical examinations were performed by physicians. Family history was obtained by genetic counselors. Serum hormones and chromosome karyotypes were detected in the hospital’s genetics and endocrinology laboratory. Skeletal X-ray and pituitary magnetic resonance imaging (MRI) were conducted in the hospital’s medical imaging center. Ultrasonography of sexual glands was performed in the hospital’s ultrasonic department.

### Molecular analysis

All patients and their parents were subjected to molecular analysis to identify the genetic basis of IHH. Genomic DNA (gDNA) was extracted from peripheral blood samples using DNeasy Blood and Tissue Kit (QIAGEN, Hilden, Germany). For patients enrolled before January 2018, their gDNA samples were first subjected to *ANOS1* gene analysis using Sanger sequencing. Subsequently, whole exome sequencing (WES) was performed for *ANOS1*-negative samples. For patients enrolled after January 2018, their gDNA samples were directly subjected to WES.

The gnomAD was employed to exclude the polymorphic alleles, and HGMD (Professional 2025.2) was engaged to confirm the known pathogenic variant. For novel variants, in-silico tools were used to predict the functional consequences, including PROVEAN, SIFT, PolyPhen-2, MutationTaster, FATHMM, NetGene2, NNSPLICE 0.9 and RNA Splicer. The pathogenicity of variants was evaluated according to the guidelines of American College of Medical Genetics (ACMG) [16].

### Treatment and follow-up

After diagnosis, all patients, except those lost to follow-up or newly diagnosed, were treated with intramuscular injection of HCG (500–1000 U) alone or in combination with human menopausal gonadotropin (HMG, 75 U) twice weekly for 6–12 weeks annually. To promote penile growth and orchiocatabasis, or to maintain secondary sex characteristics, some patients were treated with oral testosterone undecanoate (TU) at a dose of 2–3 mg/(kg·d). Only 3 patients older than 14 years of age were transitioned to pulsatile injection of GnRH (5–10 µg/90 min) via an infusion pump.

Most patients were followed up at intervals of 3–12 months, except for a few with poor compliance. The height, weight, penile length, testicular volume and related hormonal parameters were measured at every visit. The doses of medication were adjusted based on the hormonal results.

### Statistical analysis

SPSS Statistics 17.0 software (SPSS, Chicago, United States) was used to calculate means and standard deviation (SD), compare means, and evaluate differences. Student’s *t* test or One-Way ANOVA was performed to compare means for data distributed normally, whereas non-parametric Mann-Whitney *U* test or Kruskal-Wallis *H* test was engaged for data not. For cross-tables, Yates’ continuity correction or Fisher’s exact test was conducted where applicable. A statistically significant difference was defined as *p* < 0.05. The vertical scatter plots showing mean with SD were generated by GraphPad Prism 5 software (GraphPad, Boston, USA).

## Results

### Mutational spectrum

A total of 51 patients from 48 unrelated families in southern China, including three pairs of siblings, were confirmed to have pathogenic or likely pathogenic variants in the causative genes of IHH. As shown in Table 1 and Table S1, variants in *ANOS1*, *CHD7*, *FGFR1*, *HS6ST1*, *KISS1R*, *PROKR2* and *SOX11* gene accounted for 11 (11/51, 21.57%), 9 (9/51, 17.65%), 23 (23/51, 45.10%), 1 (1/51, 1.96%), 1 (1/51, 1.96%), 3 (3/51, 5.88%) and 3 (3/51, 5.88%) cases, respectively. Among them, *FGFR1* gene was the most frequent cause, followed by *ANOS1* and *CHD7* gene.

**Table 1:** Deleterious variants identified in 51 male IHH patients

Family	Patient	Gender	Gene	Variant	Nucleotide change	Amino acid change	Zygosity	Inheritance	Reported previously?	ACMG category
F1	P1	Male	*FGFR1*	c.797delCinsTT (p.Thr266Ilefs*6)	Small indel	Frameshift	Het	Paternal	Novel 1	Pathogenic
F2	P2	Male	*FGFR1*	c.376delG (p.Glu126Argfs*26)	Small deletion	Frameshift	Het	*De novo*	Novel 2	Pathogenic
F3	P3	Male	*CHD7*	c.5222G > C (p.Arg1741Pro)	Base substitution	Missense	Het	*De novo*	Known 1	Pathogenic
F4	P4	Male	*FGFR1*	8p11.23-p11.22 (36788433–38458282)del	Gross deletion	NA	Het	*De novo*	Novel 3	Pathogenic
F5	P5	Male	*FGFR1*	c.424_425delGA (p.Asp142*)	Small deletion	Nonsense	Het	*De novo*	Novel 4	Pathogenic
F6	P6	Male	*CHD7*	c.5050G > A (p.?)	Base substitution	Splicing	Het	*De novo*	Known 2	Pathogenic
F7	P7	Male	*PROKR2*	c.533G > C (p.Trp178Ser)	Base substitution	Missense	Het	Paternal	Known 3	Likely pathogenic
F8	P8	Male	*CHD7*	c.4186–2 A > G	Base substitution	Splicing	Het	*De novo*	Novel 5	Pathogenic
F9	P9	Male	*FGFR1*	8p11.23-p11.22 (38147917–38975686)del	Gross deletion	NA	Het	*De novo*	Novel 6	Pathogenic
F10	P10	Male	*CHD7*	c.3655 C > T (p.R1219*)	Base substitution	Nonsense	Het	*De novo*	Known 4	Pathogenic
F11	P11	Male	*CHD7*	c.4291 A > C (p.Lys1431Gln)	Base substitution	Missense	Het	*De novo*	Novel 7	Likely pathogenic
F12	P12	Male	*FGFR1*	c.709G > A (p.Gly237Ser)	Base substitution	Missense	Het	*De novo*	Known 5	Pathogenic
F13	P13	Male	*SOX11*	c.347 A > G (p.Tyr116Cys)	Base substitution	Missense	Het	*De novo*	Known 6	Pathogenic
F14	P14	Male	*ANOS1*	c.814 C > T (p.Arg272*)	Base substitution	Nonsense	Hemi	Maternal	Known 7	Pathogenic
F15	P15	Male	*FGFR1*	c.481 A > G (p.Met161Val)	Base substitution	Missense	Het	*De novo*	Novel 8	Likely pathogenic
F16	P16	Male	*CHD7*	c.3754T > C (p.Cys1252Arg)	Base substitution	Missense	Het	*De novo*	Novel 9	Likely pathogenic
F17	P17	Male	*FGFR1*	c.2025delG (p.Ile676Serfs*38)	Small deletion	Frameshift	Het	*De novo*	Novel 10	Pathogenic
F18	P18	Male	*FGFR1*	c.246_247delAG (p.Glu84Glyfs*26)	Small deletion	Frameshift	Het	*De novo*	Known 8	Pathogenic
F19	P19	Male	*FGFR1*	c.568T > G (p.Trp190Gly)	Base substitution	Missense	Het	*De novo*	Novel 11	Likely pathogenic
F20	P20	Male	*ANOS1*	c.1267 C > T (p.Arg423*)	Base substitution	Nonsense	Hemi	Maternal	Known 9	Pathogenic
F21	P21	Male	*CHD7*	c.5405-7G > A	Base substitution	Splicing	Het	*De novo*	Known 10	Pathogenic
F22	P22	Male	*ANOS1*	c.209delA (p.?)	Small deletion	Splicing	Hemi	Maternal	Novel 12	Pathogenic
F23	P23	Male	*SOX11*	c.87 C > A (p.Cys29*)	Base substitution	Nonsense	Het	*De novo*	Known 11	Pathogenic
F24	P24	Male	*ANOS1*	c.531_541 + 2delTCTGTACAAAGGT	Small deletion	Splicing	Hemi	Maternal	Novel 13	Pathogenic
F25	P25	Male	*FGFR1*	c.570G > T (p.Trp190Cys)	Base substitution	Missense	Het	Maternal	Novel 14	Likely pathogenic
F26	P26	Male	*FGFR1*	c.2197 A > T (p.Met733Leu)	Base substitution	Missense	Het	*De novo*	Novel 15	Likely pathogenic
F27	P27	Male	*HS6ST1*	c.1144 C > T (p.Arg382Trp)	Base substitution	Missense	Het	Maternal	Known 12	Likely pathogenic
F28	P28	Male	*FGFR1*	c.936G > A (p.Lys312=)	Base substitution	Splicing	Het	*De novo*	Known 13	Pathogenic
F29	P29	Male	*KISS1R*	c.182 C > A (p.Ser61*)	Base substitution	Nonsense	Het	Maternal	Known 14	Pathogenic
F30	P30	Male	*FGFR1*	c.565 C > T (p.Arg189Cys)	Base substitution	Missense	Het	Paternal	Known 15	Likely pathogenic
F31	P31	Male	*ANOS1*	c.1267 C > T (p.Arg423*)	Base substitution	Nonsense	Hemi	Maternal	Known 9	Pathogenic
F32	P32	Male	*PROKR2*	c.991G > A (p.Val331Met)	Base substitution	Missense	Hom	Parental	Known 16	Likely pathogenic
F33	P33	Male	*ANOS1*	c.1503_1506delTGTC (p.Val502Asnfs*46)	Small deletion	Frameshift	Hemi	Maternal	Novel 16	Pathogenic
F34	P34	Male	*FGFR1*	c.1411_1414delTGGGinsCCC (p.Trp471Profs*10)	Small indel	Frameshift	Het	*De novo*	Novel 17	Pathogenic
F35	P35	Male	*FGFR1*	c.11G > A (p.Trp4*)	Base substitution	Nonsense	Het	Maternal	Known 17	Pathogenic
F36	P36	Male	*SOX11*	c.158T > G (p.Met53Arg)	Base substitution	Missense	Het	*De novo*	Known 18	Likely pathogenic
F37	P37	Male	*FGFR1*	c.1828G > C (p.Gly610Arg)	Base substitution	Missense	Het	*De novo*	Novel 18	Likely pathogenic
F38	P38	Male	*CHD7*	c.7831–2 A > G	Base substitution	Splicing	Het	Paternal	Novel 19	Pathogenic
F39	P39	Male	*FGFR1*	c.709G > A (p.Gly237Ser)	Base substitution	Missense	Het	*De novo*	Known 5	Pathogenic
F33	P40	Male	*ANOS1*	c.1503_1506delTGTC (p.Val502Asnfs*46)	Small deletion	Frameshift	Hemi	Maternal	Novel 16	Pathogenic
F40	P41	Male	*FGFR1*	c.1429delA (p.?)	Small deletion	Splicing	Het	*De novo*	Novel 20	Pathogenic
F41	P42	Male	*ANOS1*	c.668G > A (p.Trp223*)	Base substitution	Nonsense	Hemi	Maternal	Novel 21	Pathogenic
F42	P43	Male	*CHD7*	c.3226 A > G (p.Lys1076Glu)	Base substitution	Missense	Het	*De novo*	Novel 22	Likely pathogenic
F43	P44	Male	*FGFR1*	c.1780 C > T (p.Gln594*)	Base substitution	Nonsense	Het	*De novo*	Known 19	Pathogenic
F24	P45	Male	*ANOS1*	c.531_541 + 2delTCTGTACAAAGGT	Small deletion	Splicing	Hemi	Maternal	Novel 13	Pathogenic
F44	P46	Male	*PROKR2*	c.533G > C (p.Trp178Ser)	Base substitution	Missense	Het	Paternal	Known 3	Likely pathogenic
F45	P47	Male	*FGFR1*	c.2084 C > T (p.Thr695Ile)	Base substitution	Missense	Het	*De novo*	Known 20	Pathogenic
F46	P48	Male	*FGFR1*	c.817G > A (p.Val273Met)	Base substitution	Missense	Het	Maternal	Known 21	Likely pathogenic
F47	P49	Male	*FGFR1*	c.1049 C > T (p.Ser350Phe)	Base substitution	Missense	Het	*De novo*	Novel 23	Likely pathogenic
F48	P50	Male	*ANOS1*	c.784 C > T (p.Arg262*)	Base substitution	Nonsense	Hemi	Maternal	Known 22	Pathogenic
F48	P51	Male	*ANOS1*	c.784 C > T (p.Arg262*)	Base substitution	Nonsense	Hemi	Maternal	Known 22	Pathogenic

Het, heterozygous; Hemi, hemizygous; Hom, homozygous

Of the 51 IHH patients, 39 (39/51, 76.47%) carried a heterozygous variant, 11 (11/51, 21.59%) harbored a hemizygous variant, and only 1 (1/51, 1.96%) had a homozygous variant. *De novo* deleterious variants accounted for more than half of cases (29/51, 56.86%), whereas maternal and paternal variants were responsible for 31.37% (16/51) and 9.80% (5/51) of cases respectively, and only 1 patient (1/51, 1.96%) had a homozygous variant transmitted from both parents.

Forty-five different variants, including 22 known and 23 novel variants, were disclosed without a hotspot variant. Among them, 33 (33/45, 73.33%) were base substitutions, 8 (8/45, 17.78%) were small deletions, 2 (2/45, 4.44%) were gross deletions and 2 (2/45, 4.44%) were small indels according to the nucleotide change. When corresponding to the amino acid change, 19 (19/45, 42.22%) were missense variants, 10 (10/45, 22.22%) were nonsense variants, 8 (8/45, 17.78%) altered the splicing site, 7 (7/45, 15.56%) arose to a frameshift effect, and the rest 2 gross deletions (2/45, 4.44%) were denoted as NA because it is difficult to define their roles in message RNA processing and protein translation.

### Clinical characteristics at diagnosis

The clinical, imaging and hormonal findings of 51 IHH patients at diagnosis are displayed in Table S2, Table S3 and Table S4 respectively, and summarized in Table 2.

**Table 2:** Clinical, imaging and hormonal findings of 51 male IHH patients at diagnosis (grouped by age at diagnosis)

Characteristic	Total patients (*n* = 51)	Patients < 14 years (*n* = 41)	Patients ≥ 14 years (*n* = 10)	*p* value^a^
Family history	13.73% (7/51)	9.76% (4/41)	30.00% (3/10)	0.248
Age at diagnosis (years)	7.84 ± 5.89 (*n* = 51)	5.90 ± 4.81 (*n* = 41)	15.76 ± 1.68 (*n* = 10)	0.000
Height (SDS)	-0.93 ± 1.45 (*n* = 37)	-0.99 ± 1.37 (*n* = 28)	-0.74 ± 1.76 (*n* = 9)	0.666
Weight (SDS)	-0.71 ± 1.66 (*n* = 46)	-0.92 ± 1.51 (*n* = 36)	0.07 ± 2.02 (*n* = 10)	0.095
Penile length (cm)	2.41 ± 1.03 (*n* = 45)	2.17 ± 0.92 (*n* = 36)	3.39 ± 0.86(*n* = 10)	0.001
Testicular volume (mL)	1.12 ± 0.63 (*n* = 41)	0.97 ± 0.53 (*n* = 31)	1.58 ± 0.73(*n* = 10)	0.013
Clinical features				
Growth retardation (height < -2 SD)	24.32% (9/37)	21.43% (6/28)	33.33% (3/9)	0.657
Micropenis	98.04% (50/51)	100.00% (41/41)	90.00% (9/10)	0.196
Cryptorchidism	49.02% (25/51)	51.22% (21/41)	40.00% (4/10)	0.777
Secondary sexual characteristics	0.00% (0/51)	0.00% (0/41)	0.00% (0/10)	NA
Impaired olfactory function	37.93% (11/29)	36.84% (7/19)	40.00% (4/10)	1.000
Facial anomaly	11.76% (6/51)	12.20% (5/41)	10.00% (1/10)	1.000
Ocular abnormality	9.80% (5/51)	12.20% (5/41)	0.00% (0/10)	0.569
Hearing loss	9.80% (5/51)	12.20% (5/41)	0.00% (0/10)	0.569
Laryngeal problems	3.92% (2/51)	4.88% (2/41)	0.00% (0/10)	1.000
Congenital heart disease	11.76% (6/51)	14.63% (6/41)	0.00% (0/10)	0.331
Gastroesophageal reflux	1.96% (1/51)	2.44% (1/41)	0.00% (0/10)	1.000
Unilateral absence of kidney	3.92% (2/51)	2.44% (1/41)	10.00% (1/10)	0.357
Skeletal issues	3.92% (2/51)	4.88% (2/41)	0.00% (0/10)	1.000
Developmental delay	11.76% (6/51)	14.63% (6/41)	0.00% (0/10)	0.331
Weakness	1.96% (1/51)	2.44% (1/41)	0.00% (0/10)	1.000
Enuresis	1.96% (1/51)	0.00% (0/41)	10.00% (1/10)	0.196
Imaging findings				
Delayed bone age	61.90% (13/21)	46.15% (6/13)	87.50% (7/8)	0.085
Abnormal ultrasound of sexual glands	86.84% (33/38)	82.79% (24/29)	100.00% (9/9)	0.312
Abnormal pituitary MRI	47.06% (8/17)	22.22% (2/9)	75.00% (6/8)	0.057
Gonadal hormones^b^				
Basal FSH (IU/L)	0.80 ± 0.77 (*n* = 51)	0.84 ± 0.82 (*n* = 41)	0.62 ± 0.52 (*n* = 10)	0.418
Basal LH (IU/L)	0.16 ± 0.27 (*n* = 51)	0.17 ± 0.29 (*n* = 41)	0.12 ± 0.09 (*n* = 10)	0.662
Basal T (nmol/L)	0.46 ± 0.39 (*n* = 51)	0.37 ± 0.25 (*n* = 41)	0.83 ± 0.61 (*n* = 10)	0.000
Basal AMH (ng/mL)	32.42 ± 35.52 (*n* = 41)	33.64 ± 37.51 (*n* = 34)	26.51 ± 24.94 (*n* = 7)	0.635
Basal INHB (pg/mL)	39.59 ± 42.81 (*n* = 36)	42.85 ± 46.08 (*n* = 30)	23.30 ± 11.49 (*n* = 6)	0.314
Basal LH < 1.0 IU/L	98.04% (50/51)	97.56% (40/41)	100.00% (10/10)	1.000
Basal T < 3.5 nmol/L	100.00% (51/51)	100.00% (41/41)	100.00% (10/10)	NA
Low basal AMH	48.79% (20/41)	55.89% (19/34)	14.29% (1/7)	0.093
Low basal INHB	41.67% (15/36)	30.00% (9/30)	100.00% (6/6)	0.003
Peak FSH (IU/L) after GnRH stimulation	2.60 ± 2.47 (*n* = 11)	1.56 ± 1.87 (*n* = 3)	2.99 ± 2.66 (*n* = 8)	0.421
Peak LH (IU/L) after GnRH stimulation	1.61 ± 2.29 (*n* = 11)	0.49 ± 0.53 (*n* = 3)	2.03 ± 2.58 (*n* = 8)	0.346
LH/FSH after GnRH stimulation	0.53 ± 0.34 (*n* = 11)	0.36 ± 0.05 (*n* = 3)	0.59 ± 0.38 (*n* = 8)	0.838
T after HCG stimulation (nmol/L)	2.10 ± 2.89 (*n* = 25)	2.31 ± 3.19 (*n* = 20)	1.24 ± 0.80 (*n* = 5)	0.838
AMH after HCG stimulation (ng/mL)	30.73 ± 20.74 (*n* = 23)	29.25 ± 20.74 (*n* = 19)	37.75 ± 22.20 (*n* = 4)	0.469
INHB after HCG stimulation (pg/mL)	41.80 ± 24.04 (*n* = 21)	41.89 ± 25.17 (*n* = 17)	41.41 ± 21.74 (*n* = 4)	0.972

SDS, standard deviation score; FSH, follicle-stimulating hormone; LH, luteinizing hormone; T, testosterone; AMH, anti-mullerian hormone; INHB, inhibin B; GnRH, gonadotropin-releasing hormone

^a^The *p* value comparing means between < 14y and ≥ 14y group was calculated using student’s *t* test or Mann-Whitney *U* test. The *p* value of four-fold table was calculated using Yates’ continuity correction or Fisher’s exact test

^b^Those gonadal hormones below the lower detectable limit were valued with the lower detectable limit

Of these 51 patients, 7 (7/51, 13.46%) had a family history. Their age at diagnosis varied from 0.25 to 19.75 years with a mean age of 7.84 ± 5.89 years. The mean height standard deviation score (SDS) and weight SDS were − 0.93 ± 1.45 and − 0.71 ± 1.66, respectively [17]. The majority of patients (28/37, 75.68%) had a normal height, whereas 24.32% (9/37) of cases underwent growth retardation with a height below the − 2 SD of the pediatric reference height range.

In our IHH cohort, the most common clinical feature was micropenis (50/51, 98.04%), followed by cryptorchidism (25/51, 49.02%) and impaired olfactory function (11/29, 37.93%). None of the patients had hypospadias. Despite 19.61% (10/51) of patients being older than 14 years of age, none developed secondary sexual characteristics. The mean penile length and testicular volume of our patients were 2.41 ± 1.03 cm and 1.12 ± 0.63 mL, respectively. Additional clinical signs were presented in a few patients, including facial anomaly, ocular abnormality, hearing loss, laryngeal problems, congenital heart disease, gastroesophageal reflux, unilateral absence of kidney, skeletal issues, developmental delay, weakness and enuresis.

Skeletal X-ray, ultrasonography of sexual glands and pituitary MRI were conducted in 21, 38 and 17 patients, respectively. The most frequent imaging feature was abnormal ultrasound of sexual glands (33/38, 86.84%) with bilateral inguinal testes and small testes being the most common. Delayed bone age was revealed in 61.90% of patients (13/21), while pituitary MRI findings were shown in 47.06% of patients (8/17). Notably, only morphological changes or cysts rather than substantive lesions were found by pituitary MRI as the growth hormone, thyroid-stimulation hormone, prolactin, and adrenocorticotrophic hormone of these patients were normal (data not shown).

In basal conditions, the mean hormone levels were as follows: FSH, 0.80 ± 0.77 IU/L; LH, 0.16 ± 0.27 IU/L; testosterone, 0.46 ± 0.39 nmol/L; anti-mullerian hormone (AMH), 32.42 ± 35.52 ng/mL; inhibin B (INHB), 39.59 ± 42.81 pg/mL. Most cases (50/51, 98.04%) had a low LH (< 1.0 IU/L), and all (51/51, 100.00%) had a low testosterone (< 3.5 nmol/L). According to the age-related reference ranges, low AMH and INHB were shown in 48.79% (20/41) and 41.67% (15/36) of patients respectively.

In this retrospective study, 11 patients were subjected to GnRH stimulation test. The peak levels of FSH and LH were 2.60 ± 2.47 IU/L and 1.61 ± 2.29 IU/L with a LH/FSH ratio of 0.53 ± 0.34. Moreover, HCG stimulation test was performed in 25 patients, resulting in an increase of testosterone to 2.10 ± 2.89 nmol/L, whereas AMH and INHB remained similar to baseline with a value of 30.73 ± 20.74 ng/mL and 41.80 ± 24.04 pg/mL, respectively.

### Age-phenotype correlation

Based on the age at diagnosis, the 51 patients were categorized into two groups: (1) “< 14 years” group, accounting for the majority of patients (41/51, 80.39%); (2) “≥ 14 years” group, including the remaining 10 patients (10/51, 19.61%).

Significant differences were identified in penile length, testicular volume, basal testosterone, and the proportion of patients with low basal INHB between the two groups (Table 2).

### Genotype-phenotype correlation

According to the molecular spectrum, the 51 patients were divided into 4 groups: (1) “*ANOS1*” group, consisting of 11 patients carrying an *ANOS1* variant; (2) “*CHD7*” group, involving 9 patients having a *CHD7* variant; (3) “*FGFR1*” group, comprising 23 patients with a *FGFR1* variant; (4) “other” group, including 1, 1, 3 and 3 patients harboring a *HS6ST1*, *KISS1R*, *PROKR2* and *SOX11* variant respectively, totaling 8 patients.

Despite distinct molecular bases, all groups shared typical features of micropenis and absence of secondary sexual characteristics, and exhibited comparable penile lengths and testicular volumes. Genotype-phenotype correlations were also observed: (1) Most *ANOS1* patients had a family history, impaired olfactory function, and significantly lower basal AMH. Except for unilateral absence of kidney in 2 cases, no other accompanying phenotypes were observed in *ANOS1* patients; (2) *CHD7* patients were younger at diagnosis, but the difference was not significant. CHARGE features, including growth retardation, facial anomaly, ocular abnormality, hearing loss and congenital heart disease [21], and higher basal FSH and LH appeared more frequently in *CHD7* patients (Table 3).

**Table 3:** Clinical, imaging and hormonal findings of 51 male IHH patients at diagnosis (grouped by genotype)

Characteristic	*ANOS1* patients (*n* = 11)	*CHD7* patients (*n* = 9)	*FGFR1* patients (*n* = 23)	Other patients (*n* = 8)	*p* value^a^
Family history	54.55% (6/11)	0.00% (0/9)	4.35% (1/23)	0.00% (0/8)	0.000
Age at diagnosis (years)	10.87 ± 5.64 (*n* = 11)	4.16 ± 5.57 (*n* = 9)	7.72 ± 5.88 (*n* = 23)	8.13 ± 5.11 (*n* = 8)	0.087
Height (SDS)	0.11 ± 1.16 (*n* = 7)	-2.17 ± 0.69 (*n* = 7)	-0.91 ± 1.14 (*n* = 17)	-0.75 ± 2.26 (*n* = 6)	0.025
Weight (SDS)	0.29 ± 1.65 (*n* = 10)	-2.23 ± 1.54 (*n* = 8)	-0.55 ± 1.17 (*n* = 21)	-0.86 ± 2.09 (*n* = 7)	0.009
Penile length (cm)	2.75 ± 1.11 (*n* = 10)	1.81 ± 0.86 (*n* = 8)	2.45 ± 1.02(*n* = 20)	2.53 ± 1.02(*n* = 7)	0.275
Testicular volume (mL)	1.08 ± 0.53 (*n* = 10)	0.73 ± 0.30 (*n* = 8)	1.18 ± 0.74(*n* = 18)	1.60 ± 0.55(*n* = 5)	0.088
Clinical features					
Growth retardation (height < -2 SD)	0.00% (0/7)	57.14% (4/7)	17.65% (3/17)	33.33% (2/6)	0.063
Micropenis	90.91% (10/11)	100.00% (9/9)	100.00% (23/23)	100.00% (8/8)	0.549
Cryptorchidism	54.55% (6/11)	44.44% (4/9)	47.83% (11/23)	50.00% (4/8)	1.000
Secondary sexual characteristics	0.00% (0/11)	0.00% (0/9)	0.00% (0/23)	0.00% (0/8)	NA
Impaired olfactory function	87.50% (7/8)	50.00% (1/2)	15.38% (2/13)	16.67% (1/6)	0.002
Facial anomaly	0.00% (0/11)	55.56% (5/9)	0.00% (0/23)	12.50% (1/8)	0.000
Ocular abnormality	0.00% (0/11)	44.44% (4/9)	4.35% (1/23)	0.00% (0/8)	0.007
Hearing loss	0.00% (0/11)	44.44% (4/9)	0.00% (0/23)	12.50% (1/8)	0.001
Laryngeal problems	0.00% (0/11)	22.22% (2/9)	0.00% (0/23)	0.00% (0/8)	0.050
Congenital heart disease	0.00% (0/11)	55.56% (5/9)	4.35% (1/23)	0.00% (0/8)	0.001
Gastroesophageal reflux	0.00% (0/11)	11.11% (1/9)	0.00% (0/23)	0.00% (0/8)	0.333
Unilateral absence of kidney	18.18% (2/11)	0.00% (0/9)	0.00% (0/23)	0.00% (0/8)	0.093
Skeletal issues	0.00% (0/11)	11.11% (1/9)	0.00% (0/23)	12.50% (1/8)	0.150
Developmental delay	0.00% (0/11)	33.33% (3/9)	4.35% (1/23)	25.00% (2/8)	0.032
Weakness	0.00% (0/11)	11.11% (1/9)	0.00% (0/23)	0.00% (0/8)	0.333
Enuresis	0.00% (0/11)	11.11% (1/9)	0.00% (0/23)	0.00% (0/8)	0.333
Imaging findings					
Delayed bone age	50.00% (3/6)	100.00% (2/2)	66.67% (8/12)	0.00% (0/1)	0.454
Abnormal ultrasound of sexual glands	85.71% (6/7)	100.00% (7/7)	88.24% (15/17)	71.43% (5/7)	0.708
Abnormal pituitary MRI	40.00% (2/5)	60.00% (3/5)	40.00% (2/5)	50.00% (1/2)	1.000
Gonadal hormones^c^					
Basal FSH (IU/L)	0.41 ± 0.22 (*n* = 11)	1.55 ± 1.23 (*n* = 9)	0.65 ± 0.49 (*n* = 23)	0.91 ± 0.81 (*n* = 8)	0.012
Basal LH (IU/L)	0.08 ± 0.02 (*n* = 11)	0.41 ± 0.54 (*n* = 9)	0.12 ± 0.14 (*n* = 23)	0.09 ± 0.06 (*n* = 8)	0.191
Basal T (nmol/L)	0.67 ± 0.66 (*n* = 11)	0.39 ± 0.31 (*n* = 9)	0.41 ± 0.26 (*n* = 23)	0.36 ± 0.21 (*n* = 8)	0.613
Basal AMH (ng/mL)	7.61 ± 7.70 (*n* = 7)	50.92 ± 68.67 (*n* = 8)	29.11 ± 19.54 (*n* = 20)	47.73 ± 15.36 (*n* = 6)	0.003
Basal INHB (pg/mL)	26.40 ± 12.30 (*n* = 6)	64.91 ± 75.55 (*n* = 7)	33.38 ± 35.55 (*n* = 18)	42.35 ± 18.65 (*n* = 5)	0.342
Basal LH < 1.0 IU/L	100.00% (11/11)	88.89% (8/9)	100.00% (23/23)	100.00% (8/8)	0.333
Basal T < 3.5 nmol/L	100.00% (11/11)	100.00% (9/9)	100.00% (23/23)	100.00% (8/8)	NA
Low basal AMH	71.43% (5/7)	62.50% (5/8)	40.00% (8/20)	33.33% (2/6)	0.395
Low basal INHB	50.00% (3/6)	14.29% (1/7)	55.56% (10/18)	20.00% (1/5)	0.221
Peak FSH after GnRH stimulation (IU/L)	1.23 ± 1.20 (*n* = 5)	8.95 (*n* = 1)	2.38 ± 1.05 (*n* = 4)	3.98 (*n* = 1)	0.177^b^
Peak LH after GnRH stimulation (IU/L)	0.39 ± 0.27 (*n* = 5)	7.63 (*n* = 1)	1.09 ± 0.82 (*n* = 4)	3.87 (*n* = 1)	0.107^b^
LH/FSH after GnRH stimulation	0.39 ± 0.15 (*n* = 5)	0.85 (*n* = 1)	0.51 ± 0.48 (*n* = 4)	0.97 (*n* = 1)	0.556^b^
T after HCG stimulation (nmol/L)	0.58 ± 0.27 (*n* = 4)	5.57 ± 5.38 (*n* = 4)	1.07 ± 1.19 (*n* = 12)	3.00 ± 2.45 (*n* = 5)	0.103
AMH after HCG stimulation (ng/mL)	29.22 ± 20.68 (*n* = 4)	29.78 ± 26.76 (*n* = 4)	25.25 ± 19.89 (*n* = 11)	48.28 ± 12.87 (*n* = 4)	0.689
INHB after HCG stimulation (pg/mL)	35.69 ± 10.82 (*n* = 4)	38.51 ± 21.04 (*n* = 4)	35.30 ± 23.78 (*n* = 10)	76.00 ± 18.21 (*n* = 3)	0.052

SDS, standard deviation score; FSH, follicle-stimulating hormone; LH, luteinizing hormone; T, testosterone; AMH, anti-mullerian hormone; INHB, inhibin B; GnRH, gonadotropin-releasing hormone

^a^The *p* value comparing means among 4 groups was calculated using One-Way ANOVA or Kruskal-Wallis *H* test, except for those marked with an asterisk. The *p* value of cross-table was calculated using Fisher’s exact test

^b^The *p* value comparing means between *ANOS1* and *FGFR1* group was calculated using student’s *t* test or Mann-Whitney *U* test

^c^Those gonadal hormones below the lower detectable limit were valued with the lower detectable limit

### Treatment and follow-up

Among the 51 patients, 13 were lost to follow-up, 4 were newly diagnosed without treatment, whereas 34 maintained follow-up and medication. All 34 patients were treated with HCG, including 28 (28/34, 82.35%) with HCG monotherapy and 6 (6/34, 17.65%) receiving HCG and HMG combination therapy. Seven of them (7/34, 20.59%) were further supplemented with TU, and 3 (3/34, 8.82%), P42, P50 and P51, were transitioned to pulsatile GnRH treatment.

**Fig. 1:**
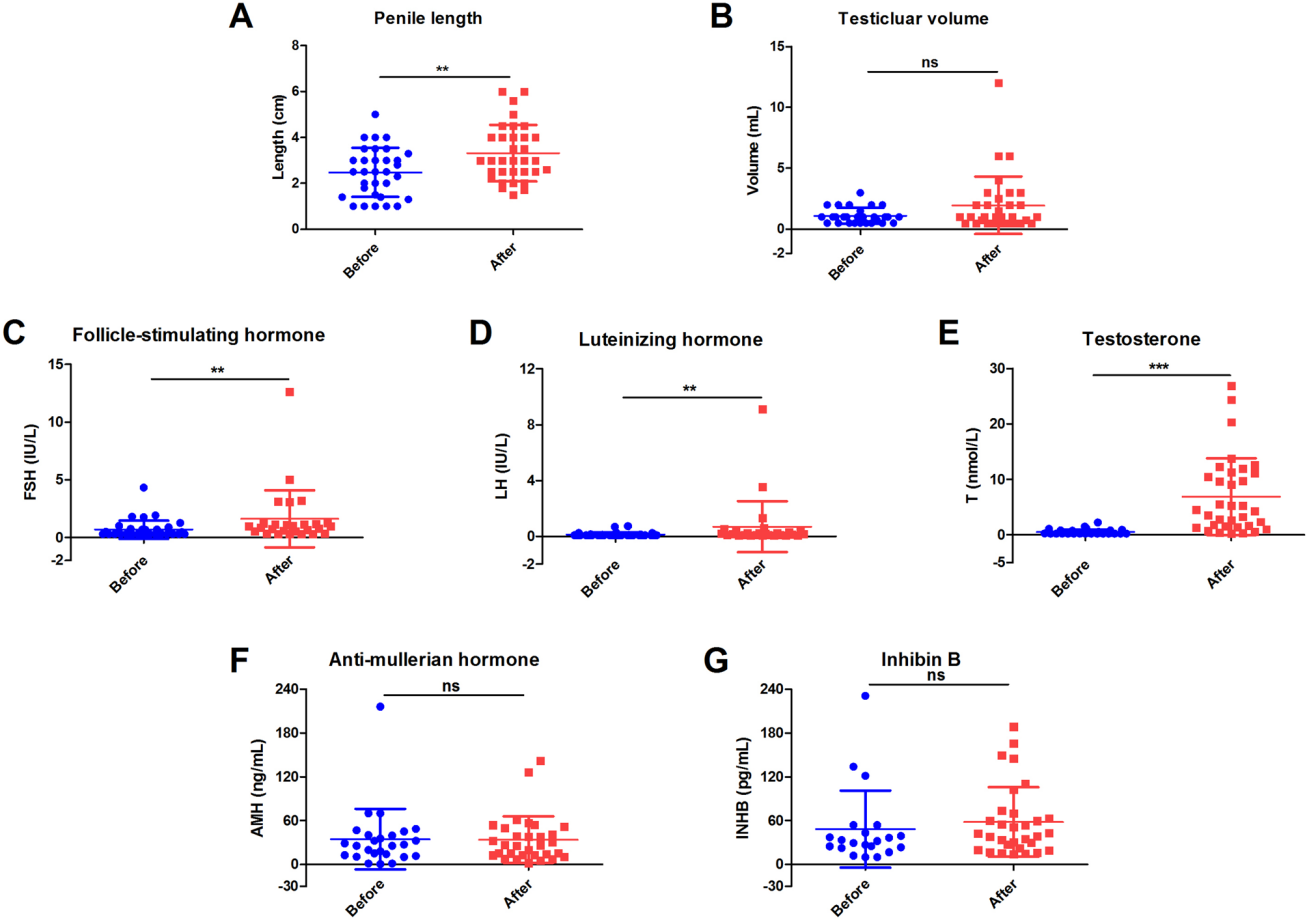
Comparison of clinical and hormonal parameters of 34 male IHH patients before and after treatment. **A** Penile length. **B** Testicular volume. **C** Follicle-stimulating hormone. **D** Luteinizing hormone. **E** Testosterone. **F** Anti-mullerian hormone. **G** Inhibin B. Results are presented as mean ± SD; ns, not significant; ** *p* < 0.01; ***p < 0.0001

Clinical follow-up lasted from 0.17 to 6.92 years with a mean time of 2.75 ± 1.82 years. After treatment, the levels of FSH, LH and testosterone elevated markedly, whereas AMH and INHB remained unchanged. Penile growth occurred in 21 patients (21/34, 61.76%), and testicular enlargement presented in 11 (11/34, 32.35%). Statistical analysis confirmed the significant increase of mean penile length, but the mean testicular volume was not significantly changed. Notably, 3 unrelated cases, P25, P48 and P50, who were administrated with HCG monotherapy, a combination therapy of HCG, HMG and TU, and GnRH therapy transitioned from HCG along with TU, respectively, showed optimal drug responses and gained the best benefits (Table 4, Table S5, Table S6 and Fig. 1).

**Table 4:** Comparison of clinical and hormonal parameters of 34 male IHH patients before and after treatment

Patient	Treatment	Follow-up time (years)	Penile length (cm)		Testicular volume (mL)		FSH (IU/L)^a^		LH (IU/L)^a^		T (nmol/L)^a^	
			Before	After	Before	After	Before	After	Before	After	Before	After
P1	HCG	1.50	1	2.5	Unmeasurable	Unmeasurable	1.76	3.05	0.12	0.24	0.38	10.45
P2	HCG	6.92	1	4	Unmeasurable	Left: 1 Right: 0.5	< 0.3	1.15	< 0.07	0.09	1.22	9.7
P4	HCG + HMG	3.16	1	3	0.5	1	< 0.3	0.33	< 0.07	0.09	0.38	9.71
P5	HCG	6.83	Unknown	2.2	Unmeasurable	0.5	0.68	1.09	< 0.07	< 0.07	0.92	0.57
P6	HCG	1.33	1	2.5	0.5	0.5	4.32	3.1	0.73	0.3	< 0.24	24.37
P7	HCG	1.67	1.4	2	Unmeasurable	0.5	0.5	0.86	< 0.07	< 0.07	< 0.24	12.59
P9	HCG	0.84	1.8	1.8	0.5	0.5	0.76	ND	< 0.07	ND	< 0.24	12.29
P11	HCG + HMG	5.83	1.3	1.7	0.5	0.5	0.4	ND	< 0.07	ND	< 0.24	9.06
P12	HCG	0.83	1.4	1.5	0.5	0.5	1.01	1.24	0.08	< 0.07	< 0.24	4.51
P15	HCG	5.25	1	2	Left: Unmeasurable Right: 1	0.5	< 0.3	ND	< 0.07	ND	0.35	3.51
P18	HCG	2.59	2.3	2.6	0.5	0.5	< 0.3	ND	0.14	ND	< 0.24	20.33
P19	HCG	0.59	3.5	3.5	2	2	0.67	0.55	0.09	0.22	< 0.24	1.59
P20	HCG	0.33	2.5	2.5	0.5	0.5	0.44	ND	0.13	ND	< 0.24	11.11
P22	HCG + TU	2.42	2	2.5	0.5	0.5	< 0.3	1.2	< 0.07	0.11	< 0.24	3.24
P24	HCG	5.00	2.5	3	Unmeasurable	Left: Unmeasurable Right: 1	< 0.3	< 0.3	< 0.07	0.18	< 0.24	0.29
P25	HCG	5.67	3	5.6	1	12	< 0.3	3.2	< 0.07	1.32	0.8	5.23
P27	HCG	1.17	2.8	3.5	1	1	1.8	ND	0.07	ND	< 0.24	13.77
P28	HCG + TU	3.17	3.3	4	1	2.5	< 0.3	1.14	0.1	0.25	< 0.24	1.61
P30	HCG + HMG	2.92	2.5	4.5	0.5	1	< 0.3	0.76	< 0.07	0.17	< 0.24	11.93
P31	HCG + HMG + TU	2.58	2.5	2.5	2	2	< 0.3	0.6	< 0.07	0.13	0.36	4.28
P33	HCG	3.75	3	3	1	1.5	< 0.3	< 0.3	< 0.07	< 0.07	< 0.24	< 0.24
P34	HCG	1.25	4	4	1.5	2	0.48	0.65	0.11	0.19	0.34	5.21
P36	HCG	1.67	1.5	3	Unmeasurable	Unmeasurable	< 0.3	0.54	< 0.07	0.11	0.83	1.01
P38	HCG	3.50	3.5	4.5	1	4	1.26	1.18	0.68	0.52	0.26	2.77
P40	HCG	4.00	2	2	1	1	< 0.3	< 0.3	< 0.07	< 0.07	0.46	1.39
P42	HCG transitioning to GnRH	1.17	5	5	2	3	< 0.3	< 0.3	< 0.07	0.1	0.49	0.38
P43	HCG	2.67	2	4.5	1	3	1.91	1.02	0.27	0.59	0.77	0.77
P45	HCG	1.75	Unknown	Unknown	Left: 1 Right: 0.5	Left: 1 Right: 0.5	< 0.3	0.35	< 0.07	< 0.07	1.09	1.82
P46	HCG + HMG + TU	3.33	3.5	4	2	3	< 0.3	0.98	0.24	0.38	0.44	2.66
P47	HCG	0.17	3	3	2	2	< 0.3	ND	< 0.07	ND	0.52	2.35
P48	HCG + HMG + TU	2.75	4	6	3	6	< 0.3	0.78	0.24	0.45	0.57	26.85
P49	HCG	1.00	3	3	1	1	0.69	0.98	< 0.07	0.2	0.45	1.33
P50	HCG + TU transitioning to GnRH	3.34	3	6	1	6	< 0.3	12.64	< 0.07	9.12	1.52	11.28
P51	HCG + TU transitioning to GnRH	2.67	4	4	1	1	0.88	5.02	< 0.07	3.56	2.22	5.5
Mean ± SD		2.75 ± 1.82	2.48 ± 1.07	3.32 ± 1.23	1.10 ± 0.65	1.95 ± 2.36	0.68 ± 0.79	1.62 ± 2.47	0.13 ± 0.15	0.69 ± 1.82	0.52 ± 0.44	6.87 ± 6.91
p value^a^		-	0.005		0.316		0.001		0.001		0.000	

FSH, follicle-stimulating hormone; LH, luteinizing hormone; T, testosterone; HCG, human chorionic gonadotropin; HMG, human menopausal gonadotropin; GnRH, gonadotropin-releasing hormone; TU, testosterone undecanoate; ND, not done

^a^The *p* value comparing means between before and after treatment group was calculated using student’s *t* test or Mann-Whitney *U* test

^b^Those gonadal hormones below the lower detectable limit were valued with the lower detectable limit

## Discussion

IHH is a set of rare diseases characterized by abnormal sexual development with clinical heterogeneity and genotypic complexity. To describe clinical and genetic features of male IHH patients in southern China and evaluate therapeutic effects of current treatments, we enrolled this cohort consisting of 51 male IHH patients.

Consistent with prior reports, our patients manifested typical clinical symptom of micropenis and absence of secondary sexual characteristics, and characteristic biochemical features of low testosterone along with low LH [3, 7]. However, the mean age at diagnosis of 7.84 ± 5.89 years in this study was much younger than previous studies [1, 2, 7, 8, 12, 18–22], probably due to the incorporation of genetic findings into case definition. IHH patients are usually not diagnosed until late adolescence or early adulthood based on clinical and biochemical manifestations. The wide use of genetic testing, especially NGS, accelerates the molecular diagnosis once the clinical diagnosis is made or suspected, suggesting that a combination of clinical, biochemical and genetic criteria will benefit early diagnosis.

In previous studies, variants in the *FGFR1*, *ANOS1*, *PROKR2*, and *CHD7* gene account for most male IHH patients [1, 2, 4, 12, 23] (Table S7). In our study, the most frequent causative gene was *FGFR1* (23/51, 45.10%), followed by *ANOS1* (11/51, 21.57%) and *CHD7* (9/51, 17.65%), revealing a similar molecular composition of male IHH between southern China and other countries or other regions of China. Twenty-three novel variants were identified in *FGFR1*, *ANOS1* and *CHD7* gene, which expands the mutational spectrum of IHH. Moreover, 8 patients were influenced by a paternal or maternal deleterious variant in the *CHD7*, *PROKR2*, *FGFR1* and *HS6ST1* gene, respectively. Although these genes can cause autosomal dominant IHH, the carrier parents were phenotypically normal and preserved fertility because of incomplete penetrance or highly phenotypic variability within families and among patients.

Age-phenotype and genotype-phenotype correlations were revealed in this study. The penile length, testicular volume and basal testosterone increased with age significantly, showing that the penis, testes and testosterone secretion could increase during puberty age, though they lacked obvious sexual development. *ANOS1* and *CHD7* patients rather than *FGFR1* patients had distinctive features. Specifically, the family history, impaired olfactory function and much lower basal AMH were hallmarks for *ANOS1* patients, whereas CHARGE phenotypes and higher basal FSH and LH could help to recognize *CHD7* patients at younger age.

As basal AMH was much lower in patients with *ANOS1* variants than other molecular causes, a differentially diagnostic value of AMH was prominent. Additionally, low basal AMH and INHB were displayed in 48.79% (20/41) and 41.67% (15/36) of our patients respectively, indicating their potential as diagnostic biomarkers for IHH, which fits well with previous studies [24–26].

After diagnosis, 34 patients were treated with HCG alone or conjugated with HMG. Among them, oral TU was added to the therapeutic schedule of 7 patients, while 3 patients were transitioned to pulsatile GnRH treatment. The treatments lasting 2.75 ± 1.82 years were effective as they markedly increased the penile length, and the levels of FSH, LH and testosterone, and yielded an increase of penile length in 21 patients (61.76%) and an enlargement of testicular volume in 11 patients (32.35%). No significant difference was shown among different therapeutic strategies. Three patients responded optimally to different strategies, highlighting the need of personalized approaches.

In fact, limitations exist in this retrospective study. As we know, GnRH stimulation test is crucial to the diagnosis of IHH. However, only 21.57% (11/51) of our patients were willing to receive GnRH stimulation test. Future work should focus on strategies to improve patient participation in GnRH stimulation test or to develop alternative diagnostic methods.

In this study, most cases were pediatric patients whose clinical phenotypes and gonadal hormone levels overlapped with those of both children with other disorders of sexual development and even normal controls. A definitive IHH diagnosis could not be made base on clinical signs and gonadal hormones without molecular evidence. Therefore, we incorporated genetic etiology as the fourth diagnostic criterion for IHH. Future work could further investigate the overall diagnostic yield of genetic testing when molecularly negative cases eventually meet definitive diagnostic criteria.

## Conclusions

Our study reports 51 male IHH patients from southern China, describes their phenotypic and genotypic characteristics, and shares our experience with the follow-up and therapeutic effects, which enriches the patient resources and clinical data. Our study reveals *FGFR1*, *ANOS1* and *CHD7* as the most common causes of IHH in southern China, and finds 23 novel variants to extend the mutational spectrum of IHH. Our study indicates that a combination of clinical, biochemical and genetic criteria will facilitate early diagnosis. Our study also suggests that the family history, impaired olfactory function, CHARGE features, and basal AMH, FSH and LH have diagnostic values in distinguishing different molecular bases.

## Supplementary Information

Below is the link to the electronic supplementary material.

Supplementary Material 1

Supplementary Material 2

Supplementary Material 3

Supplementary Material 4

Supplementary Material 5

Supplementary Material 6

Supplementary Material 7

## Data Availability

The data that support the findings of this study are available from the corresponding author upon reasonable request.

## Abbreviations

IHH: Idiopathic hypogonadotropic hypogonadism
GnRH: Gonadotropin-releasing hormone
LH: Luteinizing hormone
FSH: Follicle-stimulating hormone
HCG: Human chorionic gonadotropin
NGS: Next-generation sequencing
MRI: Magnetic resonance imaging
gDNA: Genomic DNA
ACMG: American College of Medical Genetics
WES: Whole exome sequencing
HMG: Human menopausal gonadotropin
TU: Testosterone undecanoate
SD: Sstandard deviation
SDS: Standard deviation score
AMH: Anti-mullerian hormone
INHB: Inhibin B
