# Supplementary material for: Clinical characteristics, genetic spectrum and therapeutic effects of 51 male patients with idiopathic hypogonadotropic hypogonadism from southern China
Source: Orphanet J Rare Dis. 2025 Nov 12;20:574. doi: 10.1186/s13023-025-04050-2 (PMC12613655; doi:10.1186/s13023-025-04050-2)
Supplement: Supplementary file 1 — Supplementary Material 1 [file 13023_2025_4050_MOESM1_ESM.docx]

**Table S1. ACMG category and criteria of variants identified in 51 male IHH patients**

| **Patient** | **Gene** | **Variant** | **ACMG category** | **ACMG criteria** |
| --- | --- | --- | --- | --- |
| P1 | *FGFR1* | c.797delCinsTT (p.Thr266Ilefs*6) | Pathogenic | PVS1+PM2_Supporting+PP4 |
| P2 | *FGFR1* | c.376delG (p.Glu126Argfs*26) | Pathogenic | PVS1+PS2+PM2_Supporting+PP4 |
| P3 | *CHD7* | c.5222G>C (p.Arg1741Pro) | Pathogenic | PS2+PS4_Moderate+PM1+PM2_Supporting +PP3+PP4 |
| P4 | *FGFR1* | 8p11.23-p11.22 (36788433-38458282)del | Pathogenic | PVS1+PS2+PM2_Supporting+PP4 |
| P5 | *FGFR1* | c.424_425delGA (p.Asp142*) | Pathogenic | PVS1+PS2+PM2_Supporting+PP4 |
| P6 | *CHD7* | c.5050G>A (p.?) | Pathogenic | PVS1+PS2+PS4+PM2_Supporting+PP4 |
| P7 | *PROKR2* | c.533G>C (p.Trp178Ser) | Likely pathogenic | PS3+PP3+PP4 |
| P8 | *CHD7* | c.4186-2A>G | Pathogenic | PVS1+PS2+PM2_Supporting+PP4 |
| P9 | *FGFR1* | 8p11.23-p11.22 (38147917-38975686)del | Pathogenic | PVS1+PS2+PM2_Supporting+PP4 |
| P10 | *CHD7* | c.3655C>T (p.R1219*) | Pathogenic | PVS1+PS2+PS4_Moderate+ PM2_Supporting+PP4 |
| P11 | *CHD7* | c.4291A>C (p.Lys1431Gln) | Likely pathogenic | PS2+PM2_Supporting+PP3+PP4 |
| P12 | *FGFR1* | c.709G>A (p.Gly237Ser) | Pathogenic | PS2+PS4_Moderate+PM1+PM2_Supporting+PP3+PP4 |
| P13 | *SOX11* | c.347A>G (p.Tyr116Cys) | Pathogenic | PS2+PS3+PS4+PP3+PP4 |
| P14 | *ANOS1* | c.814C>T (p.Arg272*) | Pathogenic | PVS1+PS4_Moderate+PM2_Supporting+PP4 |
| P15 | *FGFR1* | c.481A>G (p.Met161Val) | Likely pathogenic | PS2+PM2_Supporting+PM5+PP4 |
| P16 | *CHD7* | c.3754T>C (p.Cys1252Arg) | Likely pathogenic | PS2+PM2_Supporting+PP3+PP4 |
| P17 | *FGFR1* | c.2025delG (p.Ile676Serfs*38) | Pathogenic | PVS1+PS2+PM2_Supporting+PP4 |
| P18 | *FGFR1* | c.246_247delAG (p.Glu84Glyfs*26) | Pathogenic | PVS1+PS2+PS4_Moderate+PM2_Supporting+PP4 |
| P19 | *FGFR1* | c.568T>G (p.Trp190Gly) | Likely pathogenic | PS2+PM2_supporting+PP3+PP4 |
| P20 | *ANOS1* | c.1267C>T (p.Arg423*) | Pathogenic | PVS1+PS4+PM2_Supporting+PP4 |
| P21 | *CHD7* | c.5405-7G>A | Pathogenic | PS2+PS3+PS4+PM2_supporting+PP3+PP4 |
| P22 | *ANOS1* | c.209delA (p.?) | Pathogenic | PVS1+PM2_Supporting+PP4 |
| P23 | *SOX11* | c.87C>A (p.Cys29*) | Pathogenic | PVS1+PS2+PS4_Moderate+PM2_Supporting |
| P24 | *ANOS1* | c.531_541+2delTCTGTACAAAGGT | Pathogenic | PVS1+PM2_Supporting+PP1+PP4 |
| P25 | *FGFR1* | c.570G>T (p.Trp190Cys) | Likely pathogenic | PM1+PM2_supporting+PP1+PP3+PP4 |
| P26 | *FGFR1* | c.2197A>T (p.Met733Leu) | Likely pathogenic | PS2+PM2_supporting+PP3+PP4 |
| P27 | *HS6ST1* | c.1144C>T (p.Arg382Trp) | Likely pathogenic | PS3+PS4_Moderate+PP4 |
| P28 | *FGFR1* | c.936G>A (p.Lys312=) | Pathogenic | PVS1+PS2+PS4_Moderate+PM2_supporting+PP4 |
| P29 | *KISS1R* | c.182C>A (p.Ser61*) | Pathogenic | PVS1+PS4_Moderate+PM2_supporting |
| P30 | *FGFR1* | c.565C>T (p.Arg189Cys) | Likely pathogenic | PS4_Moderate+PM1+PM2_Supporting+PP4 |
| P31 | *ANOS1* | c.1267C>T (p.Arg423*) | Pathogenic | PVS1+PS4+PM2_Supporting+PP4 |
| P32 | *PROKR2* | c.991G>A (p.Val331Met) | Likely pathogenic | PS3+PM1+PP4 |
| P33 | *ANOS1* | c.1503_1506delTGTC (p.Val502Asnfs*46) | Pathogenic | PVS1+PM2_Supporting+PP1+PP4 |
| P34 | *FGFR1* | c.1411_1414delTGGGinsCCC (p.Trp471Profs*10) | Pathogenic | PVS1+PS2+PM2_Supporting+PP4 |
| P35 | *FGFR1* | c.11G>A (p.Trp4*) | Pathogenic | PVS1+PS4_Moderate+PM2_Supporting+PP4 |
| P36 | *SOX11* | c.158T>G (p.Met53Arg) | Likely pathogenic | PS2+PS4_Moderate+PM2_Supporting+PP3+PP4 |
| P37 | *FGFR1* | c.1828G>C (p.Gly610Arg) | Likely pathogenic | PS2+PM2_Supporting+PM5+PP3+PP4 |
| P38 | *CHD7* | c.7831-2A>G | Pathogenic | PVS1+PM2_Supporting+PP4 |
| P39 | *FGFR1* | c.709G>A (p.Gly237Ser) | Pathogenic | PS2+PS4_Moderate+PM1+PM2_Supporting+PP3+PP4 |
| P40 | *ANOS1* | c.1503_1506delTGTC (p.Val502Asnfs*46) | Pathogenic | PVS1+PM2_Supporting+PP1+PP4 |
| P41 | *FGFR1* | c.1429delA (p.?) | Pathogenic | PVS1+PS2+PM2_Supporting+PP4 |
| P42 | *ANOS1* | c.668G>A (p.Trp223*) | Pathogenic | PVS1+PM2_Supporting+PP4 |
| P43 | *CHD7* | c.3226A>G (p.Lys1076Glu) | Likely pathogenic | PS2+PM2_Supporting+PP3+PP4 |
| P44 | *FGFR1* | c.1780C>T (p.Gln594*) | Pathogenic | PVS1+PS2+PS4_Moderate+PM2_Supporting+PP4 |
| P45 | *ANOS1* | c.531_541+2delTCTGTACAAAGGT | Pathogenic | PVS1+PM2_Supporting+PP1+PP4 |
| P46 | *PROKR2* | c.533G>C (p.Trp178Ser) | Likely pathogenic | PS3+PP3+PP4 |
| P47 | *FGFR1* | c.2084C>T (p.Thr695Ile) | Pathogenic | PS2+PS4_Moderate+PM1+PM2_Supporting+PP3+PP4 |
| P48 | *FGFR1* | c.817G>A (p.Val273Met) | Likely pathogenic | PS4+PM1+PM2_Supporting+PP3+PP4 |
| P49 | *FGFR1* | c.1049C>T (p.Ser350Phe) | Likely pathogenic | PS2+PM1+PM2_Supporting+PP3+PP4 |
| P50 | *ANOS1* | c.784C>T (p.Arg262*) | Pathogenic | PVS1+PS4_Moderate+PM2_Supporting+PP1+PP4 |
| P51 | *ANOS1* | c.784C>T (p.Arg262*) | Pathogenic | PVS1+PS4_Moderate+PM2_Supporting+PP1+PP4 |

Het, heterozygous; Hemi, hemizygous; Hom, homozygous.
