# Supplementary material for: Clinical characteristics, genetic spectrum and therapeutic effects of 51 male patients with idiopathic hypogonadotropic hypogonadism from southern China
Source: Orphanet J Rare Dis. 2025 Nov 12;20:574. doi: 10.1186/s13023-025-04050-2 (PMC12613655; doi:10.1186/s13023-025-04050-2)
Supplement: Supplementary file 2 — Supplementary Material 2 [file 13023_2025_4050_MOESM2_ESM.docx]

**Table S2. Clinical features of 51 male IHH patients at diagnosis**

| **Family** | **Patient** | **Family history** | **Age at diagnosis (years)** | **Height (SDS)****†** | **Weight (SDS)†** | **Penile length (cm)** | **Testicular**  **volume**  **(mL)** | **Micropenis** | **Cryptorchidism** | **Secondary sexual features** | **Olfactory function*** | **Other clinical signs** |
| --- | --- | --- | --- | --- | --- | --- | --- | --- | --- | --- | --- | --- |
| F1 | P1 | - | 0.25 | -0.3 | +0.3 | 1 | Unmeasurable | + | + | - | ND | - |
| F2 | P2 | - | 0.33 | -2.0 | -3.0 | 1 | Unmeasurable | + | + | - | ND | - |
| F3 | P3 | - | 0.33 | -2.5 | -3.7 | 2 | 1 | + | - | - | ND | Congenital heart disease, visual impairment, hearing loss |
| F4 | P4 | - | 0.42 | -0.7 | -0.7 | 1 | 0.5 | + | + | - | ND | - |
| F5 | P5 | - | 0.42 | Unknown | -0.5 | Unknown | Unmeasurable | + | + | - | ND | - |
| F6 | P6 | - | 0.42 | -1.5 | -0.7 | 1 | 0.5 | + | - | - | ND | Cleft palate, congenital heart disease, exotropia, hearing loss, developmental delay |
| F7 | P7 | - | 0.50 | -2.5 | -1.6 | 1.4 | Unmeasurable | + | + | - | ND | - |
| F8 | P8 | - | 0.50 | Unknown | -3.0 | 2 | 0.3 | + | + | - | ND | Low-set ears, low hairlines, webbed neck, facial abnormality, hearing loss, congenital heart disease, widely spaced nipples, motor delay |
| F9 | P9 | - | 0.58 | -0.3 | +0.8 | 1.8 | 0.5 | + | + | - | ND | Patent foramen ovale, esotropia |
| F10 | P10 | - | 0.58 | -3.0 | -4.5 | Unknown | Unknown | + | - | - | ND | Nystagmus, ear malformations, hearing loss, congenital heart disease, congenital laryngomalacia, gastroesophageal reflux, developmental delay |
| F11 | P11 | - | 0.67 | -2.1 | -2.3 | 1.3 | 0.5 | + | + | - | ND | - |
| F12 | P12 | - | 1.25 | Unknown | -0.2 | 1.4 | 0.5 | + | + | - | ND | - |
| F13 | P13 | - | 1.33 | -3.8 | -3.6 | 1.5 | Unmeasurable | + | + | - | ND | Microtia, congenital aural atresia, hearing loss, global developmental delay |
| F14 | P14 | - | 1.42 | Unknown | -0.1 | 1 | 1 | + | + | - | ND | - |
| F15 | P15 | - | 1.75 | -1.3 | +0.1 | 1 | Left: Unmeasurable  Right: 1 | + | + | - | ND | Mental retardation |
| F16 | P16 | - | 2.33 | -1.8 | -0.8 | 0.7 | Left: 0.5  Right: Unmeasurable | + | + | - | ND | Low-set ears, esotropia, high-arched palate, congenital heart disease, congenital laryngeal stridor |
| F17 | P17 | - | 4.08 | -0.1 | +0.5 | 1.6 | Left: 0.5  Right: 1 | + | - | - | ND | - |
| F18 | P18 | - | 4.33 | -0.5 | -1.1 | 2.3 | 0.5 | + | - | - | ND | - |
| F19 | P19 | - | 4.33 | -0.1 | -0.3 | 3.5 | 2 | + | - | - | ND | - |
| F20 | P20 | - | 4.92 | -1.0 | -0.3 | 2.5 | 0.5 | + | - | - | ND | - |
| F21 | P21 | - | 5.67 | -3.0 | -2.7 | 2 | 1 | + | - | - | ND | Protruding ears, lower extremity weakness |
| F22 | P22 | - | 5.75 | -0.3 | +0.3 | 2 | 0.5 | + | + | - | ND | - |
| F23 | P23 | - | 6.08 | +1.2 | +0.5 | 3.5 | 1 | + | + | - | Normal | - |
| F24 | P24 | + | 6.25 | Unknown | -1.5 | 2.5 | Unmeasurable | + | + | - | Impaired | - |
| F25 | P25 | + | 6.83 | -0.9 | -1.6 | 3 | 1 | + | - | - | Normal | - |
| F26 | P26 | - | 8.33 | Unknown | Unknown | Unknown | Unknown | + | + | - | Impaired | - |
| F27 | P27 | - | 9.33 | +1.5 | +1.1 | 2.8 | 1 | + | - | - | Normal | - |
| F28 | P28 | - | 9.58 | +0.4 | -1.0 | 3.3 | 1 | + | - | - | Normal | - |
| F29 | P29 | - | 9.92 | +1.0 | +1.9 | Unknown | 2 | + | - | - | Normal | - |
| F30 | P30 | - | 10.58 | -1.8 | -0.3 | 2.5 | 0.5 | + | - | - | Normal | - |
| F31 | P31 | - | 11.00 | +1.3 | +1.1 | 2.5 | 2 | + | - | - | Normal | Absence of the left kidney |
| F32 | P32 | - | 11.08 | Unknown | Unknown | 3.5 | 2 | + | - | - | Impaired | - |
| F33 | P33 | + | 11.58 | Unknown | Unknown | 3 | 1 | + | - | - | Impaired | - |
| F34 | P34 | - | 11.75 | Unknown | -0.7 | 4 | 1.5 | + | - | - | Normal | - |
| F35 | P35 | - | 11.83 | -1.7 | -1.6 | 2.5 | 0.5 | + | + | - | Normal | - |
| F36 | P36 | - | 11.83 | Unknown | -3.0 | 1.5 | Unmeasurable | + | + | - | Normal | Intellectual disability, dysarthria, brachydactyly of the fifth finger |
| F37 | P37 | - | 12.00 | Unknown | 0.3 | Unknown | Unknown | + | + | - | Normal | - |
| F38 | P38 | - | 12.42 | Unknown | Unknown | 3.5 | 1 | + | + | - | Impaired | - |
| F39 | P39 | - | 12.50 | -0.6 | -1.2 | 2.5 | 1 | + | - | - | Impaired | - |
| F33 | P40 | + | 13.17 | -1.3 | -0.1 | 2 | 1 | + | - | - | Impaired | - |
| F40 | P41 | - | 13.83 | Unknown | Unknown | 3.5 | 2 | + | - | - | Normal | - |
| F41 | P42 | - | 14.08 | -0.5 | +0.2 | 5 | 2 | - | + | - | Impaired | - |
| F42 | P43 | - | 14.50 | -1.3 | -0.2 | 2 | 1 | + | - | - | Normal | Paralysis of the right face, facial asymmetry, enuresis |
| F43 | P44 | - | 14.67 | -2.2 | -2.2 | 3 | 2 | + | - | - | Normal | - |
| F24 | P45 | + | 14.83 | Unknown | -1.2 | Unknown | Left: 1  Right: 0.5 | + | + | - | Impaired | Absence of the right kidney |
| F44 | P46 | - | 15.00 | -1.9 | -1.3 | 3.5 | 2 | + | - | - | Normal | - |
| F45 | P47 | - | 15.33 | -2.5 | +0.4 | 3 | 2 | + | + | - | Normal | - |
| F46 | P48 | - | 15.75 | -2.6 | -1.8 | 4 | 3 | + | - | - | Normal | - |
| F47 | P49 | - | 16.83 | +1.7 | +2.3 | 3 | 1 | + | - | - | Normal | - |
| F48 | P50 | + | 16.83 | +1.6 | 0 | 3 | 1 | + | + | - | Impaired | - |
| F48 | P51 | + | 19.75 | +1.0 | +4.5 | 4 | 1 | + | - | - | Impaired | - |

SDS, standard deviation score; ND, not done.

† The height and weight were evaluated in terms of the reference chart for Chinese children and adolescents from birth to 18 years of age [17].

* The olfactory function of patients before 6 years of age was not evaluated as the younger children could not formulate smell clearly.
