# Supplementary material for: Clinical characteristics, genetic spectrum and therapeutic effects of 51 male patients with idiopathic hypogonadotropic hypogonadism from southern China
Source: Orphanet J Rare Dis. 2025 Nov 12;20:574. doi: 10.1186/s13023-025-04050-2 (PMC12613655; doi:10.1186/s13023-025-04050-2)
Supplement: Supplementary file 3 — Supplementary Material 3 [file 13023_2025_4050_MOESM3_ESM.docx]

**Table S3. Imaging features of 51 male IHH patients at diagnosis**

| **Patient** | **Age at diagnosis (years)** | **Bone age** | **Ultrasonography of sexual glands** | **Pituitary MRI** |
| --- | --- | --- | --- | --- |
| P1 | 0.25 | ND | Bilateral inguinal testes | ND |
| P2 | 0.33 | ND | Bilateral inguinal testes | ND |
| P3 | 0.33 | ND | ND | ND |
| P4 | 0.42 | ND | Bilateral inguinal testes | ND |
| P5 | 0.42 | ND | Bilateral inguinal testes, small testes | ND |
| P6 | 0.42 | ND | ND | No abnormality |
| P7 | 0.50 | ND | Bilateral inguinal testes | ND |
| P8 | 0.50 | ND | Small testes | ND |
| P9 | 0.58 | ND | Bilateral inguinal testes, small testes | ND |
| P10 | 0.58 | ND | Hydrocele of the bilateral testes | No abnormality |
| P11 | 0.67 | ND | Bilateral sliding testes | Small pituitary |
| P12 | 1.25 | ND | Bilateral inguinal testes | ND |
| P13 | 1.33 | ND | Bilateral inguinal testes | No abnormality |
| P14 | 1.42 | ND | Bilateral inguinal testes | ND |
| P15 | 1.75 | ND | Dysgenesis of the left testis, hydrocele of the right spermatic cord | No abnormality |
| P16 | 2.33 | ND | Bilateral inguinal testes, hypoplastic testes | Small adenohypophysis |
| P17 | 4.08 | ND | ND | ND |
| P18 | 4.33 | Delayed | ND | ND |
| P19 | 4.33 | ND | Small testes | ND |
| P20 | 4.92 | ND | ND | No abnormality |
| P21 | 5.67 | ND | Small testes | ND |
| P22 | 5.75 | Age-appropriate | ND | ND |
| P23 | 6.08 | ND | Bilateral sliding testes | ND |
| P24 | 6.25 | ND | Bilateral celiac testes, small testes | ND |
| P25 | 6.83 | Age-appropriate | ND | No abnormality |
| P26 | 8.33 | ND | ND | ND |
| P27 | 9.33 | Age-appropriate | No abnormality | ND |
| P28 | 9.58 | Age-appropriate | No abnormality | ND |
| P29 | 9.92 | ND | No abnormality | ND |
| P30 | 10.58 | Delayed | Small testes | ND |
| P31 | 11.00 | Age-appropriate | No abnormality | ND |
| P32 | 11.08 | ND | ND | ND |
| P33 | 11.58 | ND | ND | ND |
| P34 | 11.75 | Age-appropriate | No abnormality | ND |
| P35 | 11.83 | ND | ND | ND |
| P36 | 11.83 | ND | Bilateral inguinal testes, hypoplastic testes | ND |
| P37 | 12.00 | Age-appropriate | Small testes | ND |
| P38 | 12.42 | Delayed | Bilateral sliding testes | ND |
| P39 | 12.50 | Delayed | Small testes | ND |
| P40 | 13.17 | Delayed | ND | No abnormality |
| P41 | 13.83 | Delayed | Bilateral testicular microlithiasis | ND |
| P42 | 14.08 | Delayed | Bilateral sliding testes | Rathke’s cyst |
| P43 | 14.50 | Delayed | Small testes | Small pituitary |
| P44 | 14.67 | Delayed | ND | ND |
| P45 | 14.83 | ND | Bilateral inguinal testes | ND |
| P46 | 15.00 | ND | Small testes | Small pituitary |
| P47 | 15.33 | Delayed | Right inguinal testis | Rathke’s cyst, small pineal cyst |
| P48 | 15.75 | Delayed | Small testes | No abnormality |
| P49 | 16.83 | Delayed | Small testes | Small pituitary |
| P50 | 16.83 | Delayed | Left inguinal testis, small testes | Small pituitary |
| P51 | 19.75 | Age-appropriate | Small testes | No abnormality |

ND, not done.
