# Supplementary material for: Clinical characteristics, genetic spectrum and therapeutic effects of 51 male patients with idiopathic hypogonadotropic hypogonadism from southern China
Source: Orphanet J Rare Dis. 2025 Nov 12;20:574. doi: 10.1186/s13023-025-04050-2 (PMC12613655; doi:10.1186/s13023-025-04050-2)
Supplement: Supplementary file 4 — Supplementary Material 4 [file 13023_2025_4050_MOESM4_ESM.docx]

**Table S4. Gonadal hormones of 51 male IHH patients at diagnosis**

| **Patient** | **Age at diagnosis**  **(years)** | **Baseline** | | | | | **GnRH stimulation** | | | **HCG stimulation** | | |
| --- | --- | --- | --- | --- | --- | --- | --- | --- | --- | --- | --- | --- |
|  |  | **FSH**  **(IU/L)** | **LH**  **(IU/L)** | **T**  **(nmol/L)** | **AMH**  **(****ng/mL)** | **INHB**  **(pg/mL)** | **FSH**  **(IU/L)** | **LH**  **(IU/L)** | **LH/FSH** | **T**  **(nmol/L)** | **AMH**  **(ng/mL)** | **INHB**  **(pg/mL)** |
| ***Reference range*** | | 0.26-3* | 0.02-0.3* | <0.24-0.45* | 1) 0-1 y: 76.2-268.87;  2) 2-5 y: 46.67-233.01;  3) 6-9 y: 28.43-164.47;  4) 10-14 y: 2.22-126.13;  5) 15-18 y: 2.01-32.48;  6) ≥18 y: 1.45-18.77 | 1) 0-5 y: 10-602.34;  2) 5-10 y: 21-166;  3) 10-14 y: 41-328;  4) 14-18 y: 135-368;  5) ≥18 y: 15-295 |  |  |  |  |  |  |
| P1 | 0.25 | 1.76 | 0.12 | 0.38 | 44.99 | 121.35 | ND | ND | ND | ND | ND | ND |
| P2 | 0.33 | <0.3 | <0.07 | 1.22 | 25.42 | ND | ND | ND | ND | ND | ND | ND |
| P3 | 0.33 | 1.85 | 1.67 | 1.08 | ND | ND | ND | ND | ND | ND | ND | ND |
| P4 | 0.42 | <0.3 | <0.07 | 0.38 | 48.62 | 133.89 | ND | ND | ND | ND | ND | ND |
| P5 | 0.42 | 0.68 | <0.07 | 0.92 | ND | ND | ND | ND | ND | ND | ND | ND |
| P6 | 0.42 | 4.32 | 0.73 | <0.24 | 216.11 | 230.79 | ND | ND | ND | ND | ND | ND |
| P7 | 0.50 | 0.5 | <0.07 | <0.24 | 32.38 | <10 | ND | ND | ND | 0.63 | 46.46 | ND |
| P8 | 0.50 | 0.69 | <0.07 | <0.24 | 27.76 | 36.41 | ND | ND | ND | 10.69 | 66.06 | 62.53 |
| P9 | 0.58 | 0.76 | <0.07 | <0.24 | 40.4 | 29.34 | ND | ND | ND | 0.57 | 30.12 | 47.17 |
| P10 | 0.58 | 2.09 | 0.07 | <0.24 | 56.37 | 67.31 | ND | ND | ND | ND | ND | ND |
| P11 | 0.67 | 0.4 | <0.07 | <0.24 | 26.97 | 32.03 | ND | ND | ND | 1.66 | 19.6 | 38.79 |
| P12 | 1.25 | 1.01 | 0.08 | <0.24 | 17.95 | 16.78 | ND | ND | ND | 0.87 | 11.7 | 21.44 |
| P13 | 1.33 | <0.3 | <0.07 | <0.24 | 48.01 | 47.32 | ND | ND | ND | ND | ND | ND |
| P14 | 1.42 | 0.82 | <0.07 | <0.24 | 5.14 | 10.91 | ND | ND | ND | ND | ND | ND |
| P15 | 1.75 | <0.3 | <0.07 | 0.35 | 12.55 | ND | ND | ND | ND | 0.59 | 9.12 | ND |
| P16 | 2.33 | 1.13 | <0.07 | <0.24 | 13.17 | 23.68 | ND | ND | ND | 9.67 | 30.65 | 41.44 |
| P17 | 4.08 | <0.3 | <0.07 | <0.24 | 14.08 | 13.09 | ND | ND | ND | 0.83 | 32.54 | 10 |
| P18 | 4.33 | <0.3 | 0.14 | <0.24 | 11.58 | 43.29 | ND | ND | ND | 0.3 | 16.85 | 55.72 |
| P19 | 4.33 | 0.67 | 0.09 | <0.24 | ND | ND | ND | ND | ND | <0.24 | 2.81 | 11.89 |
| P20 | 4.92 | 0.44 | 0.13 | <0.24 | 15.06 | 11.94 | ND | ND | ND | 0.71 | 22.15 | 25.73 |
| P21 | 5.67 | <0.3 | <0.07 | <0.24 | 2.25 | <10 | ND | ND | ND | <0.24 | 2.81 | 11.26 |
| P22 | 5.75 | <0.3 | <0.07 | <0.24 | 1.11 | 38.92 | ND | ND | ND | <0.24 | 5.55 | 29 |
| P23 | 6.08 | <0.3 | <0.07 | <0.24 | 50.92 | 55.9 | ND | ND | ND | 5.26 | 54.17 | 90.37 |
| P24 | 6.25 | <0.3 | <0.07 | <0.24 | 0.42 | ND | ND | ND | ND | ND | ND | ND |
| P25 | 6.83 | <0.3 | <0.07 | 0.8 | ND | ND | ND | ND | ND | ND | ND | ND |
| P26 | 8.33 | <0.3 | <0.07 | <0.24 | 50.63 | 22.11 | ND | ND | ND | ND | ND | ND |
| P27 | 9.33 | 1.8 | 0.07 | <0.24 | 70.12 | 53.79 | ND | ND | ND | 5.93 | 61.26 | 82.11 |
| P28 | 9.58 | <0.3 | 0.1 | <0.24 | 32.38 | 25 | ND | ND | ND | ND | ND | ND |
| P29 | 9.92 | 2.12 | 0.09 | 0.4 | 56.2 | 44.76 | ND | ND | ND | ND | ND | ND |
| P30 | 10.58 | <0.3 | <0.07 | <0.24 | 13.97 | 24.84 | ND | ND | ND | 4.53 | 43.18 | 78.73 |
| P31 | 11.00 | <0.3 | <0.07 | 0.36 | 19.81 | 36.52 | <0.3 | 0.11 | 0.37 | 0.87 | 54.6 | 49.87 |
| P32 | 11.08 | 1.72 | <0.07 | <0.24 | ND | ND | ND | ND | ND | ND | ND | ND |
| P33 | 11.58 | <0.3 | <0.07 | <0.24 | ND | ND | ND | ND | ND | ND | ND | ND |
| P34 | 11.75 | 0.48 | 0.11 | 0.34 | 35.41 | 23.51 | ND | ND | ND | ND | ND | ND |
| P35 | 11.83 | <0.3 | 0.07 | <0.24 | 6.19 | <10 | ND | ND | ND | 0.29 | 6.5 | 25.99 |
| P36 | 11.83 | <0.3 | <0.07 | 0.83 | ND | ND | ND | ND | ND | 0.97 | ND | ND |
| P37 | 12.00 | 1.25 | 0.21 | 0.62 | 10.68 | 18.41 | ND | ND | ND | ND | ND | ND |
| P38 | 12.42 | 1.26 | 0.68 | 0.26 | 39.31 | 54.12 | ND | ND | ND | ND | ND | ND |
| P39 | 12.50 | 1.06 | <0.07 | <0.24 | 27.19 | 12.56 | 3.71 | 1.1 | 0.30 | 1.08 | 39.71 | 30.09 |
| P40 | 13.17 | <0.3 | <0.07 | 0.46 | 10.61 | ND | 0.67 | 0.27 | 0.40 | ND | ND | ND |
| P41 | 13.83 | 2.01 | 0.72 | 0.34 | 59.99 | 26.87 | ND | ND | ND | ND | ND | ND |
| P42 | 14.08 | <0.3 | <0.07 | 0.49 | ND | 26.76 | 3.06 | 0.7 | 0.23 | 0.48 | 34.57 | 38.17 |
| P43 | 14.50 | 1.91 | 0.27 | 0.77 | 25.42 | ND | 8.95 | 7.63 | 0.85 | ND | ND | ND |
| P44 | 14.67 | 0.91 | <0.07 | <0.24 | 3.64 | <10 | ND | ND | ND | ND | ND | ND |
| P45 | 14.83 | <0.3 | <0.07 | 1.09 | 1.09 | ND | ND | ND | ND | ND | ND | ND |
| P46 | 15.00 | <0.3 | 0.24 | 0.44 | 28.75 | ND | 3.98 | 3.87 | 0.97 | 2.22 | 31.24 | 55.53 |
| P47 | 15.33 | <0.3 | <0.07 | 0.52 | 46.79 | 22.53 | 1.31 | 0.3 | 0.23 | 0.78 | ND | ND |
| P48 | 15.75 | <0.3 | 0.24 | 0.57 | 69.82 | 37.18 | 1.82 | 2.22 | 1.22 | 2 | 68.85 | 59.93 |
| P49 | 16.83 | 0.69 | <0.07 | 0.45 | 10.06 | <10 | 2.67 | 0.74 | 0.28 | 0.73 | 16.34 | 11.99 |
| P50 | 16.83 | <0.3 | <0.07 | 1.52 | ND | 33.34 | 1.83 | 0.62 | 0.34 | ND | ND | ND |
| P51 | 19.75 | 0.88 | <0.07 | 2.22 | ND | ND | <0.3 | 0.19 | 0.63 | ND | ND | ND |

FSH, follicle-stimulating hormone; LH, luteinizing hormone; T, testosterone; AMH, anti-mullerian hormone; INHB, inhibin B; GnRH, gonadotropin-releasing hormone; ND, not done.

* The reference ranges of FSH, LH and T were pre-pubertal values.
