# Supplementary material for: Clinical characteristics, genetic spectrum and therapeutic effects of 51 male patients with idiopathic hypogonadotropic hypogonadism from southern China
Source: Orphanet J Rare Dis. 2025 Nov 12;20:574. doi: 10.1186/s13023-025-04050-2 (PMC12613655; doi:10.1186/s13023-025-04050-2)
Supplement: Supplementary file 5 — Supplementary Material 5 [file 13023_2025_4050_MOESM5_ESM.docx]

**Table S5. Clinical and hormonal parameters of 51 male IHH patients after treatment**

| **Patient** | **Treatment** | **Age at last visit (years)** | **Follow-up time (years)** | **Penile length (cm)** | **Testicular volume (mL)** | **FSH**  **(IU/L)** | **LH**  **(IU/L)** | **T**  **(nmol/L)** | **AMH**  **(****ng/mL)** | **INHB**  **(pg/mL)** |
| --- | --- | --- | --- | --- | --- | --- | --- | --- | --- | --- |
| P1 | HCG | 1.75 | 1.50 | 2.5 | Unmeasurable | 3.05 | 0.24 | 10.45 | 56.85 | 149.43 |
| P2 | HCG | 7.25 | 6.92 | 4 | Left: 1  Right: 0.5 | 1.15 | 0.09 | 9.7 | 5.56 | 14.85 |
| P3 | Lost to follow-up | - | - | - | - | - | - | - | - | - |
| P4 | HCG+HMG | 3.58 | 3.16 | 3 | 1 | 0.33 | 0.09 | 9.71 | 126.23 | 69.9 |
| P5 | HCG | 7.25 | 6.83 | 2.2 | 0.5 | 1.09 | <0.07 | 0.57 | 15.35 | 17.01 |
| P6 | HCG | 1.75 | 1.33 | 2.5 | 0.5 | 3.1 | 0.3 | 24.37 | 141.6 | 145.15 |
| P7 | HCG | 2.17 | 1.67 | 2 | 0.5 | 0.86 | <0.07 | 12.59 | 32.44 | 55.03 |
| P8 | Newly diagnosed without treatment | - | - | - | - | - | - | - | - | - |
| P9 | HCG | 1.42 | 0.84 | 1.8 | 0.5 | ND | ND | 12.29 | 51.66 | 59.9 |
| P10 | Lost to follow-up | - | - | - | - | - | - | - | - | - |
| P11 | HCG+HMG | 6.50 | 5.83 | 1.7 | 0.5 | ND | ND | 9.06 | 26.28 | 63.33 |
| P12 | HCG | 2.08 | 0.83 | 1.5 | 0.5 | 1.24 | <0.07 | 4.51 | 49.8 | 38.13 |
| P13 | Lost to follow-up | - | - | - | - | - | - | - | - | - |
| P14 | Newly diagnosed without treatment | - | - | - | - | - | - | - | - | - |
| P15 | HCG | 7.00 | 5.25 | 2 | 0.5 | ND | ND | 3.51 | 25.31 | 29.63 |
| P16 | Lost to follow-up | - | - | - | - | - | - | - | - | - |
| P17 | Lost to follow-up | - | - | - | - | - | - | - | - | - |
| P18 | HCG | 6.92 | 2.59 | 2.6 | 0.5 | ND | ND | 20.33 | 37.84 | 42.06 |
| P19 | HCG | 4.92 | 0.59 | 3.5 | 2 | 0.55 | 0.22 | 1.59 | 10.5 | 15.55 |
| P20 | HCG | 5.25 | 0.33 | 2.5 | 0.5 | ND | ND | 11.11 | 37.73 | 34.73 |
| P21 | Lost to follow-up | - | - | - | - | - | - | - | - | - |
| P22 | HCG+TU | 8.17 | 2.42 | 2.5 | 0.5 | 1.2 | 0.11 | 3.24 | 6.99 | 18.88 |
| P23 | Lost to follow-up | - | - | - | - | - | - | - | - | - |
| P24 | HCG | 11.25 | 5.00 | 3 | Left: Unmeasurable  Right: 1 | <0.3 | 0.18 | 0.29 | 15.14 | 16.27 |
| P25 | HCG | 12.50 | 5.67 | 5.6 | 12 | 3.2 | 1.32 | 5.23 | 7.16 | 165.59 |
| P26 | Lost to follow-up | - | - | - | - | - | - | - | - | - |
| P27 | HCG | 10.50 | 1.17 | 3.5 | 1 | ND | ND | 13.77 | 41.17 | 73.74 |
| P28 | HCG+TU | 12.75 | 3.17 | 4 | 2.5 | 1.14 | 0.25 | 1.61 | 12.91 | 51.1 |
| P29 | Lost to follow-up | - | - | - | - | - | - | - | - | - |
| P30 | HCG+HMG | 13.50 | 2.92 | 4.5 | 1 | 0.76 | 0.17 | 11.93 | 53.75 | 102.66 |
| P31 | HCG+HMG+TU | 13.58 | 2.58 | 2.5 | 2 | 0.6 | 0.13 | 4.28 | 61.01 | 110.52 |
| P32 | Lost to follow-up | - | - | - | - | - | - | - | - | - |
| P33 | HCG | 15.33 | 3.75 | 3 | 1.5 | <0.3 | <0.07 | <0.24 | 19.8 | 14.02 |
| P34 | HCG | 13.00 | 1.25 | 4 | 2 | 0.65 | 0.19 | 5.21 | 54.01 | 30.39 |
| P35 | Lost to follow-up | - | - | - | - | - | - | - | - | - |
| P36 | HCG | 13.50 | 1.67 | 3 | Unmeasurable | 0.54 | 0.11 | 1.01 | 14.05 | 27.53 |
| P37 | Newly diagnosed without treatment | - | - | - | - | - | - | - | - | - |
| P38 | HCG | 15.92 | 3.50 | 4.5 | 4 | 1.18 | 0.52 | 2.77 | 15.44 | 53.47 |
| P39 | Newly diagnosed without treatment | - | - | - | - | - | - | - | - | - |
| P40 | HCG | 17.17 | 4.00 | 2 | 1 | <0.3 | <0.07 | 1.39 | 13.12 | 19.64 |
| P41 | Lost to follow-up | - | - | - | - | - | - | - | - | - |
| P42 | HCG transitioning to GnRH | 15.25 | 1.17 | 5 | 3 | <0.3 | 0.1 | 0.38 | 38.43 | 43.09 |
| P43 | HCG | 17.17 | 2.67 | 4.5 | 3 | 1.02 | 0.59 | 0.77 | 12.74 | 59.52 |
| P44 | Lost to follow-up | - | - | - | - | - | - | - | - | - |
| P45 | HCG | 16.58 | 1.75 | Unknown | Left: 1  Right: 0.5 | 0.35 | <0.07 | 1.82 | ND | ND |
| P46 | HCG+HMG+TU | 18.33 | 3.33 | 4 | 3 | 0.98 | 0.38 | 2.66 | ND | ND |
| P47 | HCG | 15.50 | 0.17 | 3 | 2 | ND | ND | 2.35 | 30.14 | 38.41 |
| P48 | HCG+HMG+TU | 18.50 | 2.75 | 6 | 6 | 0.78 | 0.45 | 26.85 | 26.59 | 188.69 |
| P49 | HCG | 17.83 | 1.00 | 3 | 1 | 0.98 | 0.2 | 1.33 | 2.01 | 22.31 |
| P50 | HCG+TU transitioning to GnRH | 20.17 | 3.34 | 6 | 6 | 12.64 | 9.12 | 11.28 | ND | ND |
| P51 | HCG+TU transitioning to GnRH | 22.42 | 2.67 | 4 | 1 | 5.02 | 3.56 | 5.5 | 7.98 | 33.61 |

FSH, follicle-stimulating hormone; LH, luteinizing hormone; T, testosterone; AMH, anti-mullerian hormone; INHB, inhibin B; HCG, human chorionic gonadotropin; HMG, human menopausal gonadotropin; TU, testosterone undecanoate; GnRH, gonadotropin-releasing hormone; ND, not done.
