# Supplementary material for: Clinical characteristics, genetic spectrum and therapeutic effects of 51 male patients with idiopathic hypogonadotropic hypogonadism from southern China
Source: Orphanet J Rare Dis. 2025 Nov 12;20:574. doi: 10.1186/s13023-025-04050-2 (PMC12613655; doi:10.1186/s13023-025-04050-2)
Supplement: Supplementary file 6 — Supplementary Material 6 [file 13023_2025_4050_MOESM6_ESM.docx]

**Table S6.** **Comparison of AMH and INHB of 34 male IHH patients before and after treatment**

| **Patient** | **Treatment** | **Follow-up time (years)** | **AMH (****ng/mL)** | | **INHB (pg/mL)*** | |
| --- | --- | --- | --- | --- | --- | --- |
|  |  |  | Before | After | Before | After |
| P1 | HCG | 1.50 | 44.99 | 56.85 | 121.35 | 149.43 |
| P2 | HCG | 6.92 | 25.42 | 5.56 | ND | 14.85 |
| P4 | HCG+HMG | 3.16 | 48.62 | 126.23 | 133.89 | 69.9 |
| P5 | HCG | 6.83 | ND | 15.35 | ND | 17.01 |
| P6 | HCG | 1.33 | 216.11 | 141.6 | 230.79 | 145.15 |
| P7 | HCG | 1.67 | 32.38 | 32.44 | <10 | 55.03 |
| P9 | HCG | 0.84 | 40.4 | 51.66 | 29.34 | 59.9 |
| P11 | HCG+HMG | 5.83 | 26.97 | 26.28 | 32.03 | 63.33 |
| P12 | HCG | 0.83 | 17.95 | 49.8 | 16.78 | 38.13 |
| P15 | HCG | 5.25 | 12.55 | 25.31 | ND | 29.63 |
| P18 | HCG | 2.59 | 11.58 | 37.84 | 43.29 | 42.06 |
| P19 | HCG | 0.59 | ND | 10.5 | ND | 15.55 |
| P20 | HCG | 0.33 | 15.06 | 37.73 | 11.94 | 34.73 |
| P22 | HCG+TU | 2.42 | 1.11 | 6.99 | 38.92 | 18.88 |
| P24 | HCG | 5.00 | 0.42 | 15.14 | ND | 16.27 |
| P25 | HCG | 5.67 | ND | 7.16 | ND | 165.59 |
| P27 | HCG | 1.17 | 70.12 | 41.17 | 53.79 | 73.74 |
| P28 | HCG+TU | 3.17 | 32.38 | 12.91 | 25 | 51.1 |
| P30 | HCG+HMG | 2.92 | 13.97 | 53.75 | 24.84 | 102.66 |
| P31 | HCG+HMG+TU | 2.58 | 19.81 | 61.01 | 36.52 | 110.52 |
| P33 | HCG | 3.75 | ND | 19.8 | ND | 14.02 |
| P34 | HCG | 1.25 | 35.41 | 54.01 | 23.51 | 30.39 |
| P36 | HCG | 1.67 | ND | 14.05 | ND | 27.53 |
| P38 | HCG | 3.50 | 39.31 | 15.44 | 54.12 | 53.47 |
| P40 | HCG | 4.00 | 10.61 | 13.12 | ND | 19.64 |
| P42 | HCG transitioning to GnRH | 1.17 | ND | 38.43 | 26.76 | 43.09 |
| P43 | HCG | 2.67 | 25.42 | 12.74 | ND | 59.52 |
| P45 | HCG | 1.75 | 1.09 | ND | ND | ND |
| P46 | HCG+HMG+TU | 3.33 | 28.75 | ND | ND | ND |
| P47 | HCG | 0.17 | 46.79 | 30.14 | 22.53 | 38.41 |
| P48 | HCG+HMG+TU | 2.75 | 69.82 | 26.59 | 37.18 | 188.69 |
| P49 | HCG | 1.00 | 10.06 | 2.01 | <10 | 22.31 |
| P50 | HCG+TU transitioning to GnRH | 3.34 | ND | ND | 33.34 | ND |
| P51 | HCG+TU transitioning to GnRH | 2.67 | ND | 7.98 | ND | 33.61 |
| **Mean ± SD** | | 2.75 ± 1.82 | 34.27 ± 40.59 | 34.04 ± 32.27 | 52.97 ± 55.67 | 55.16 ± 45.27 |
| ***p* value†** | | - | 0.981 | | 0.877 | |

AMH, anti-mullerian hormone; INHB, inhibin B; HCG, human chorionic gonadotropin; HMG, human menopausal gonadotropin; TU, testosterone undecanoate; GnRH, gonadotropin-releasing hormone; ND, not done; SD, standard deviation.

†The *p* value comparing means between before and after treatment group was calculated using student’s *t* test.

*Those gonadal hormones below the lower detectable limit were valued with the lower detectable limit.
