# Supplementary material for: Clinical characteristics, genetic spectrum and therapeutic effects of 51 male patients with idiopathic hypogonadotropic hypogonadism from southern China
Source: Orphanet J Rare Dis. 2025 Nov 12;20:574. doi: 10.1186/s13023-025-04050-2 (PMC12613655; doi:10.1186/s13023-025-04050-2)
Supplement: Supplementary file 7 — Supplementary Material 7 [file 13023_2025_4050_MOESM7_ESM.docx]

**Table S7.** **Comparison of the** **frequency of** **causative genes in different IHH cohorts**

| Reference | Cho CY et al [1] | Wang Y et al [2] | Liu Q et al [12] | Tommiska J et al [23] | Federici S [24] | Our study |
| --- | --- | --- | --- | --- | --- | --- |
| Region | Taiwan | Eastern China | Eastern China | Denmark | Italy | Southern China |
| Male patients with genetic findings (n) | 13 | 12 | 23 | 12 | 90 | 51 |
| Frequency of different causative genes | | | | | | |
| *ANOS1* [n (%)] | 3 (23.08%) | 2 (16.67%) | 4 (17.39%) | 4 (33.33%) | 14 (15.56%) | 11 (21.57%) |
| *CHD7* [n (%)] | 2 (15.38%) | 2 (16.67%) | 1 (4.35%) | 2 (16.67%) | 9 (10.00%) | 9 (17.65%) |
| *FEZF1* [n (%)] | 0 (0.00%) | 0 (0.00%) | 0 (0.00%) | 0 (0.00%) | 2 (2.22%) | 0 (0.00%) |
| *FGF8* [n (%)] | 0 (0.00%) | 0 (0.00%) | 0 (0.00%) | 0 (0.00%) | 2 (2.22%) | 0 (0.00%) |
| *FGFR1* [n (%)] | 4 (30.77%) | 3 (25.00%) | 9 (39.13%) | 5 (41.67%) | 18 (20.00%) | 23 (45.10%) |
| *FLRT3* [n (%)] | 0 (0.00%) | 0 (0.00%) | 0 (0.00%) | 0 (0.00%) | 1 (1.11%) | 0 (0.00%) |
| *GNRH1* [n (%)] | 0 (0.00%) | 0 (0.00%) | 0 (0.00%) | 0 (0.00%) | 2 (2.22%) | 0 (0.00%) |
| *GNRHR* [n (%)] | 0 (0.00%) | 0 (0.00%) | 0 (0.00%) | 1 (8.33%) | 7 (7.78%) | 0 (0.00%) |
| *HESX1* [n (%)] | 0 (0.00%) | 0 (0.00%) | 0 (0.00%) | 0 (0.00%) | 2 (2.22%) | 0 (0.00%) |
| *HS6ST1* [n (%)] | 1 (7.69%) | 0 (0.00%) | 0 (0.00%) | 0 (0.00%) | 1 (1.11%) | 1 (1.96%) |
| *KISS1R* [n (%)] | 0 (0.00%) | 2 (16.67%) | 0 (0.00%) | 0 (0.00%) | 6 (6.67%) | 1 (1.96%) |
| *PROK2* [n (%)] | 0 (0.00%) | 0 (0.00%) | 2 (8.70%) | 0 (0.00%) | 5 (5.56%) | 0 (0.00%) |
| *PROKR2* [n (%)] | 1 (7.69%) | 1 (8.33%) | 6 (26.09%) | 0 (0.00%) | 8 (8.89%) | 3 (5.88%) |
| *SEMA3A* [n (%)] | 0 (0.00%) | 0 (0.00%) | 1 (4.35%) | 0 (0.00%) | 3 (3.33%) | 0 (0.00%) |
| *SEMA3E* [n (%)] | 0 (0.00%) | 1 (8.33%) | 0 (0.00%) | 0 (0.00%) | 0 (0.00%) | 0 (0.00%) |
| *SOX10* [n (%)] | 0 (0.00%) | 1 (8.33%) | 0 (0.00%) | 0 (0.00%) | 3 (3.33%) | 0 (0.00%) |
| *SOX11* [n (%)] | 2 (15.38%) | 0 (0.00%) | 0 (0.00%) | 0 (0.00%) | 0 (0.00%) | 3 (5.88%) |
| *SPRY4* [n (%)] | 0 (0.00%) | 0 (0.00%) | 0 (0.00%) | 0 (0.00%) | 3 (3.33%) | 0 (0.00%) |
| *TACR3* [n (%)] | 0 (0.00%) | 0 (0.00%) | 0 (0.00%) | 0 (0.00%) | 4 (4.44%) | 0 (0.00%) |

The red color shows the most frequent causative genes in the cohort.
